# A novel and safe small molecule enhances hair follicle regeneration by facilitating metabolic reprogramming

**DOI:** 10.1038/s12276-018-0185-z

**Published:** 2018-12-06

**Authors:** Myung Jin Son, Jae Kap Jeong, Youjeong Kwon, Jae-Sung Ryu, Seon Ju Mun, Hye Jin Kim, Sung-wuk Kim, Sanghee Yoo, Jiae Kook, Hongbum Lee, Janghwan Kim, Kyung-Sook Chung

**Affiliations:** 1 Stem Cell Research Center, Korea Research Institute of Bioscience and Biotechnology (KRIBB), 125 Gwahak-ro, Yuseong-gu, Daejeon 34141 Republic of Korea; 2 Department of Functional Genomics, Korea University of Science & Technology (UST), 217 Gajungro, Yuseong-gu, Daejeon 34113 Republic of Korea; 3 HanAll Biopharma, Bongeunsaro114-gil 12, 9th Floor, Kangnam-gu Seoul, Republic of Korea; 4 ImmunoMet Therapeutics Inc., JLABS at Texas Medical Center, 2450 Holcombe Blvd, Houston, TX 77021 USA; 5 Biomedical Translational Research Center, KRIBB, 125 Gwahak-ro, Yuseong-gu, Daejeon 34141 Republic of Korea; 6 Present Address: SCAS-BTT Bioanalysis Co., Ltd, Ochang Scientific Complex 53, Yengudanji-ro, Ochang-eup, Cheongwon-gu, Cheongju-si, Chungcheongbuk-do 28115 Republic of Korea; 7 Present Address: Center for Biomolecular Sciences, University of Illinois at Chicago, 900 South Ashland Ave. 3018, Chicago, IL 60607 USA; 8 Present Address: Eco-Friendly and New Materials Research Center, Korea Research Institute of Chemical Technology, Daejeon, 34114 Republic of Korea

**Keywords:** Regeneration, Reprogramming

## Abstract

Targeting hair follicle regeneration has been investigated for the treatment of hair loss, and fundamental studies investigating stem cells and their niche have been described. However, knowledge of stem cell metabolism and the specific regulation of bioenergetics during the hair regeneration process is currently insufficient. Here, we report the hair regrowth-promoting effect of a newly synthesized novel small molecule, IM176OUT05 (IM), which activates stem cell metabolism. IM facilitated stemness induction and maintenance during an induced pluripotent stem cell generation process. IM treatment mildly inhibited mitochondrial oxidative phosphorylation and concurrently increased glycolysis, which accelerated stemness induction during the early phase of reprogramming. More importantly, the topical application of IM accelerated hair follicle regeneration by stimulating the progression of the hair follicle cycle to the anagen phase and increased the hair follicle number in mice. Furthermore, the stem cell population with a glycolytic metabotype appeared slightly earlier in the IM-treated mice. Stem cell and niche signaling involved in the hair regeneration process was also activated by the IM treatment during the early phase of hair follicle regeneration. Overall, these results show that the novel small molecule IM promotes tissue regeneration, specifically in hair regrowth, by restructuring the metabolic configuration of stem cells.

## Stem cells: A remedy to revitalize the roots

A compound that establishes metabolic conditions favorable for sustaining stem cells may also offer a safe drug for promoting hair regrowth. Drugs that inhibit mitochondrial activity help lock stem cells into a pluripotent state that allows them to actively divide and repair various tissues, but many of these drugs are toxic. Researchers led by Myung Jin Son of the Korea Research Institute of Bioscience and Biotechnology, Daejeon and ImmunoMet, USA have identified a new compound that safely achieves the same effect. This potential drug helped promote stemness in diverse stem cell types, including the highly proliferative cells that comprise hair follicles. Topical application proved more effective at promoting hair regrowth in female mice than the baldness drug minoxidil, and matched its performance in males, demonstrating its potency as a stem cell modulator.

## Introduction

Hair is produced in the hair follicle, which is a regenerating tissue that cycles through the three phases of growth (anagen), regression (catagen), and resting (telogen)^1^. Hair follicle stem cells capable of proliferation and differentiation are responsible for the cyclic regeneration process^2^, and many studies investigating stem cells, homeostasis, and the regeneration of the mammalian epidermis have been performed for hair loss control^3–6^. The hair follicle is among the most proliferative tissues in the body^7^ and undergoes repeated cycles of stem cell self-renewal and differentiation throughout life; thus, the process of hair growth requires higher bioenergetic capacities^8,9^. Accumulating evidence has shown that human hair follicle stem cells have an aerobic glycolytic metabotype^10,11^ and that cellular metabolism switches to mitochondrial-dependent oxidative phosphorylation (OXPHOS) upon differentiation^12,13^, which similarly occurs in other stem/progenitor cells, such as neural stem cells (NSCs), mesenchymal stem cells, and satellite cells^14^. Although the significance of mitochondrial biogenesis and function in hair follicle regeneration has been emphasized^15,16^, the possible applications of the metabolic control of hair follicle stem cells in hair regrowth are limited.

Induced pluripotent stem cell (iPSC) technology is a process to convert the cell fate of adult somatic cells to an embryonic stem cell (ESC)-like state by the ectopic expression of defined pluripotency-associated genes, such as OCT4, SOX2, KLF4, c-MYC (OSKM), LIN28, and NANOG^17^. This somatic cell reprograming is an inversely recapitulating process performed to turn back the developmental clock. During the initial stage of reprogramming, drastic cellular and molecular changes in genetic, epigenetic, and mitochondrial metabolic modifications occur^18^, including the substantial transformation of the mitochondrial structure into an immature phenotype and change towards mitochondria-independent glycolytic metabolism^19,20^. Importantly, this dedifferentiation process could occur both in vitro and in vivo. Recent advances in this field have provided data suggesting that in vivo partial reprogramming by the transient overexpression of the Yamanaka reprogramming factors (OSKM) promoted tissue regeneration in old mice^21^. The cyclic induction of OSKM in muscle promoted muscle regeneration by inducing the intrinsic regenerative functions of muscle stem cells following injury in aged mice. More specifically, the cyclic in vivo expression of reprogramming factors increased the epidermal and dermal thickness concomitantly with increasing Oct4 and Sox2 expression and the number of keratin 15 (K15)-positive hair follicle stem cells in progeria mouse skin^21^. Additionally, Lin28, which is an iPSC reprogramming factor^22^, has shown tissue repair capacity in some adult tissues, such as hair follicles and ear skin^8^. Lin28 transgenic mice displayed promoted cell proliferation in hair follicles and improved hair regrowth^8^. Therefore, the factors and conditions that control the acquisition and maintenance of stemness in iPSC generation could be used to boost tissue regeneration, including hair regrowth.

We previously demonstrated that optimized subtoxic doses of canonical mitochondrial inhibitors can fuel reprogramming to pluripotency by facilitating the glycolytic metabolic transition during iPSC generation^23^. Thus, we hypothesized that these inhibitors that could activate stemness and glycolytic reprogramming facilitate the cycle of hair follicle regeneration. However, mitochondrial inhibitors, such as rotenone, 2-thenoyltrifluoroacetone (TTFA), antimycin A, and potassium cyanide (KCN) (specific inhibitors of the electron transport chain (ETC)), oligomycin A (a direct ATP synthase inhibitor), and carbonyl cyanide 4-(trifluoromethoxy) phenylhydrazone (FCCP; an uncoupler), are all toxic materials^23^. Therefore, we newly synthesized a novel and safe small molecule that could inhibit mitochondrial function for further therapeutic applications, including tissue regeneration, oncology, fibrosis, and inflammatory diseases. This molecule was developed based on the characteristics of metformin, a biguanide diabetes drug that has been reported to have pleiotropic functions by expanding the NSC pool, promoting neurogenesis^24,25^ and improving the healthspan and lifespan of mice by mildly inhibiting the complex I activity of the ETC^26,27^.

Here, we report that IM176OUT05 (IM) is an optimized and improved biguanide with high solubility, greater potency and bioavailability, and better distribution that could be a candidate drug to improve tissue regeneration. The optimal low dose of IM improves the acquisition and maintenance of stem cell pluripotency in both mouse and human cell systems. Stemness markers are potently induced by IM treatment, which slightly inhibits OXPHOS and increases the production of lactate during the early stage of the reprogramming process. IM further promotes in vivo hair follicle regrowth by accelerating anagen progression through increasing glycolytic stem cell populations and further expanding highly proliferating progenitors. Therefore, we propose that IM is a candidate drug for hair regrowth that could facilitate the reprogramming of cellular metabolism.

## Materials and methods

### Reagents

IM176OUT05 (IM) is synthesized as a derivative of biguanide with improved potency and pharmacokinetic properties. Rotenone was purchased from Santa Cruz Biotechnology (Dallas, Texas, USA). Phenformin, oligomycin A, FCCP, and 4’,6-diamidino-2-phenylindole (DAPI) were purchased from Sigma (St. Louis, MO, USA). Minoxidil was purchased from Hyundai Pharm (Seoul, Republic of Korea). Metformin was purchased from Cayman Chemical (Ann Arbor, MI, USA).

### Cell culture

All animal experiments were approved by the Bioethics Committee of KRIBB. A549 (human lung carcinoma), SK-MEL-28 (human melanoma), SK-OV-3 (human ovarian cancer), 786-O (renal carcinoma), and MDA-MB-435, MDA-MB-231, and MCF-7 (human breast cancer) cells were purchased from ATCC (Manassas, VA, USA) and were maintained in RPMI (Invitrogen, Grand Island, NY, USA) supplemented with 5% fetal bovine serum (FBS, Invitrogen) and 1% penicillin/streptomycin (PS; Invitrogen). Mouse embryonic fibroblasts (MEFs) were isolated from embryonic day 12.5 embryos from CF1 mice (Laboratory Animal Resource Center, Chungcheongbuk-do, Republic of Korea) and were cultured in Dulbecco’s modified Eagle’s medium (DMEM; Invitrogen) containing 10% FBS, 0.1 mM β-mercaptoethanol (β-ME; Sigma), 1% nonessential amino acids (NEAA; Invitrogen), and 1% PS. Human foreskin fibroblasts (HFFs; ATCC) were maintained in DMEM supplemented with 10% FBS, 0.1 mM β-ME, 1% NEAA, 1% l-glutamine (Invitrogen), and 1% PS. The mouse ESC (mESC) line J1 (ATCC) was maintained on γ-irradiated MEFs or Matrigel™ (BD Biosciences, Franklin Lakes, NJ, USA)-coated plates in DMEM containing 15% FBS, 0.1 mM β-ME, 1% NEAA, 1% l-glutamine, 20 mM HEPES (Invitrogen), 1% PS, and 1000 U/ml of leukemia inhibitory factor (LIF) (Millipore, Billerica, MA, USA). The human ESC (hESC) line H9 (WiCell Research Institute, Madison, WI, USA) was routinely maintained on γ-irradiated MEFs in hESC culture medium (unconditioned medium; UM) or Matrigel™-coated plates in MEF-CM (conditioned medium) as previously described^28^.

### Virus production and iPSC generation

GP2-293 packaging cells (Clontech, Mountain View, CA, USA) were transfected with the pMX vectors harboring the human complementary DNAs (cDNAs) for Oct4 (POU5F1), Sox2, Klf4, and c-Myc (Addgene, Cambridge, MA, USA) and the VSV-G envelope vector using Lipofectamine 2000 transfection reagent (Invitrogen). Virus-containing supernatants were concentrated by ultracentrifugation (Beckman Coulter, Brea, CA, USA) at 25,000 rpm (rotor: SW32Ti) for 90 min. To generate iPSCs, MEFs or HFFs were seeded at 1 × 10^5^ cells per well in six-well plates and then were transduced with virus at a multiplicity of infection of 1 in the presence of 8 μg/ml of polybrene (Sigma) on day 1. The MEFs or HFFs were trypsinized at day 4 or 5 after seeding, respectively, and were reseeded at a density of 3 × 10^4^ cells per well in Matrigel-coated 12-well plates. On the next day, the medium was replaced with mESC or hESC medium with or without test chemicals, and the medium was changed every other day thereafter.

### Alkaline phosphatase (AP) staining

AP staining was performed with a commercially available kit according to the manufacturer’s instructions (Sigma). The cells were fixed with a fixation solution for 30 s and then were stained with an AP staining solution for 15 min in the dark. Images of AP^+^ colonies were obtained using an HP Scanjet G4010 (Hewlett-Packard, Palo Alto, CA, USA). The colony number and density were analyzed using ImageJ software.

### Oxygen consumption rate (OCR)/extracellular acidification rate (ECAR) measurement

A549 cells were treated in triplicate with serially diluted IM for 24 h and were washed prior to the OCR measurements. In the reprogramming experiments, OSKM-transduced MEFs were reseeded in triplicate at a density of 3 × 10^3^ cells per well in poly-l-lysine (Sigma)-coated 96-well XF plates (Agilent, Santa Clara, California, USA) 4 days after reprogramming. On the following day (day 5), the medium was replaced with mESC medium with or without the test chemicals. On day 7, OCR/ECAR was measured using a Seahorse XFe96 Flux analyzer according to the manufacturer’s instructions. The probe cartridge was calibrated without CO_2_ for 1 h, and then basal OCR/ECAR measurement was performed. The following ETC-targeting compounds were sequentially added at each indicated time point: 1.5 μM oligomycin (ATP synthase, complex V, inhibitor), 5 μM FCCP (uncoupler), and 0.5 μM rotenone (complex I inhibitor) + antimycin A (complex III inhibitor). The value was normalized against the cell number.

### Lactate and ATP assays

For the lactate assay, 10 μg of total protein from each treated group was used for the reaction according to the manufacturer’s protocol for the Lactate Assay Kit (BioVision, Milpitas, CA, USA). After 30 min of incubation at room temperature (RT), the absorbance was measured using a SpectraMax microplate reader (Molecular Devices, Sunnyvale, CA, USA). For the ATP assay, 0.1 μg of total protein from each treated group was used, and the ATP concentration was quantified using an ADP/ATP Ratio Assay Kit (Abcam, Cambridge, MA, USA). The luminescence was measured using a SpectraMax microplate reader (Molecular Devices).

### RNA extraction and real-time polymerase chain reaction (PCR)

Total RNA was obtained from each treated group using an RNeasy Mini Kit (Qiagen, Valencia, CA, USA). cDNA was synthesized using a SuperScript First-strand Synthesis System Kit (Invitrogen) and then was mixed using Fast SYBR® Green Master Mix (Applied Biosystems, Waltham, MA, USA). Quantitative real-time PCR was performed using a 7500 Fast Real-Time PCR System (Applied Biosystems). A list of primer sequences used in this study is presented in Supporting Information Table 1.

### Chromatin immunoprecipitation (ChIP) assay

On day 7 of reprogramming, the cells were fixed with formaldehyde to cross link histones to DNA, and then ChIP assays were performed using a SimpleChIP® Enzymatic Chromatin IP Kit (Cell Signaling Technology, Danvers, MA, USA) according to the manufacturer’s instructions. DNA was purified from precipitated immunocomplexes and was analyzed by real-time PCR using specific primers for the *Oct4* and *Nanog* promoters. Nonimmunoprecipitated total chromatin (input samples) was used as a control. The primers and antibodies used are presented in Supporting Information Tables 1 and 2, respectively.

### Immunocytochemistry

The cells were fixed with 4% paraformaldehyde for 10 min at RT, permeabilized in 0.1% Triton X-100 (Sigma) for 30 min at RT and blocked with 4% bovine serum albumin for 2 h at RT. Next, the samples were stained with the respective primary antibodies at 4 ℃ overnight and were washed with 0.05% Tween-20 (Sigma) in phosphate-buffered saline (PBS). The samples were incubated with Alexa Fluor®-conjugated secondary antibodies (Thermo Fisher Scientific) for 40 min at RT, and then florescence images were captured under an Olympus microscope (Olympus, Tokyo, Japan). The antibodies used are listed in Supporting Information Table 2.

### Hair regeneration model

Dorsal skin hairs in the telogen phase from 7-week-old C57BL/6 mice^1^ (Dae han BioLink, Chungbuk, Republic of Korea) were depilated with an animal clipper and wax (Veet, Oxy Reckitt Benckiser, Seoul, Republic of Korea). The following day, 200 μl of placebo control, 1% IM, 1% minoxidil, or 1% metformin were applied daily to the area with a sterilized cotton swab. Images of each animal were captured daily, and the level of pigmentation was quantified by the intensity of the darkness of the back color in the same area (1.6 × 3 cm) using ImageJ software. The mice were sacrificed, and skin tissues were obtained on days 0, 7, 14, and 20. Half of the tissue was used for RNA isolation, and the other half of the tissue was fixed with 4% paraformaldehyde overnight for histochemistry.

### Histological analysis

The fixed tissues were immersed in 30% sucrose and then were embedded in organic cation transporter (OCT) compound (Sakura Finetek USA Inc., Torrance, CA, USA). The frozen sections were obtained by cryostat sectioning (Leica, Wetzlar, Germany) and were stained with hematoxylin (Sigma) and eosin (Sigma) (H&E) or the respective antibodies listed in Supporting Information Table 2. Immunohistochemistry was performed as previously described^29^, and florescence images were acquired under an Olympus microscope (Olympus).

### Quantitative histomorphometry

To quantify the hair cycle, individual hair follicles in photomicrographs of H&E-stained longitudinal sections of each mouse (*n* > 10) were classified based on guidelines for the accurate classification of hair cycle stages^1^. The percentage of hair follicles in specific anagen stages was calculated in each group. Hair follicles in the same area (1300 μm width) were counted in photomicrographs of H&E-stained transverse sections of each mouse (*n* > 10).

### Fluorescence-activated cell sorting (FACS) analysis

The mice were perfused with PBS, and their skin tissue was harvested and cut into small pieces. The tissues were digested with Trypsin (Thermo Fisher Scientific) for 150 min at 37 °C and then were filtered through a 75 μm nylon mesh, followed by 30 μm mesh (BD Biosciences). The single cells were fixed, permeabilized, and blocked according to the immunostaining protocol. The cells were stained with specific antibodies (Supporting Information Table 2) and were analyzed by BD Accuri^TM^ C6 (BD Biosciences).

### Ear hole punch assay

For pinnal tissue repair assays, three holes of 2 mm in diameter in each ear were punched with ear punches (Fine Science Tools, British Columbia, Canada). The indicated concentration of IM was applied with a sterilized cotton swab every day. The hole area was measured with digital calipers (VWR, Radnor, PA, USA).

### IM penetration assay

The SK-OV-3, MDA-MB-435, 786-O, MDA-MB-231, and MCF-7 cells were seeded at 500,000 cells per well in 12-well plates. The cells were treated with 10 μM of biguanides for 30 min. The cells in each well were washed with cold PBS, and the amount of biguanides were measured using liquid chromatography tandem mass spectrometry (LC-MS/MS) (Agilent 1200 series HPLC system; Agilent 6430 MS/MS system; Agilent), and the data were analyzed using MassHunter B 01.03. For the AMP-activated protein kinase (AMPK) activation assay, 500,000 MCF-7 cells/well were plated in six-well plates. The cells were treated with concentrations ranging from 3 μM to 10 mM of biguanides and were incubated for 12 h. The cells were treated with 1% Triton X-100 cell lysis buffer, and the supernatant was collected after centrifugation at 12,000 rpm for 20 min. The protein samples (25 μg each) were added to the p-AMPK ELISA plates (Thermo Fisher Scientific), and the absorbances of samples were measured at 450 nm.

### Statistical analysis

All figures show representative data of more than three independent biological replicates. The animal experiments were independently repeated six times with *n* > 5 in each treated group. The graphs represent means ± SE; quintuplicate samples were used for the OCR/ECAR analysis; quadruplicate samples were used for the lactate assays; triplicate samples were used for AP staining, PCR analysis, and pinnal wound area analysis; and duplicate samples were used for the ATP assay (technical replicates). Student’s *t*-test was used to evaluate the intergroup comparisons, and *p* < 0.05 indicated statistical significance.

## Results

### The novel and safe small molecule IM176OUT05 (IM) improves the acquisition and maintenance of stem cell pluripotency

**Fig. 1:**
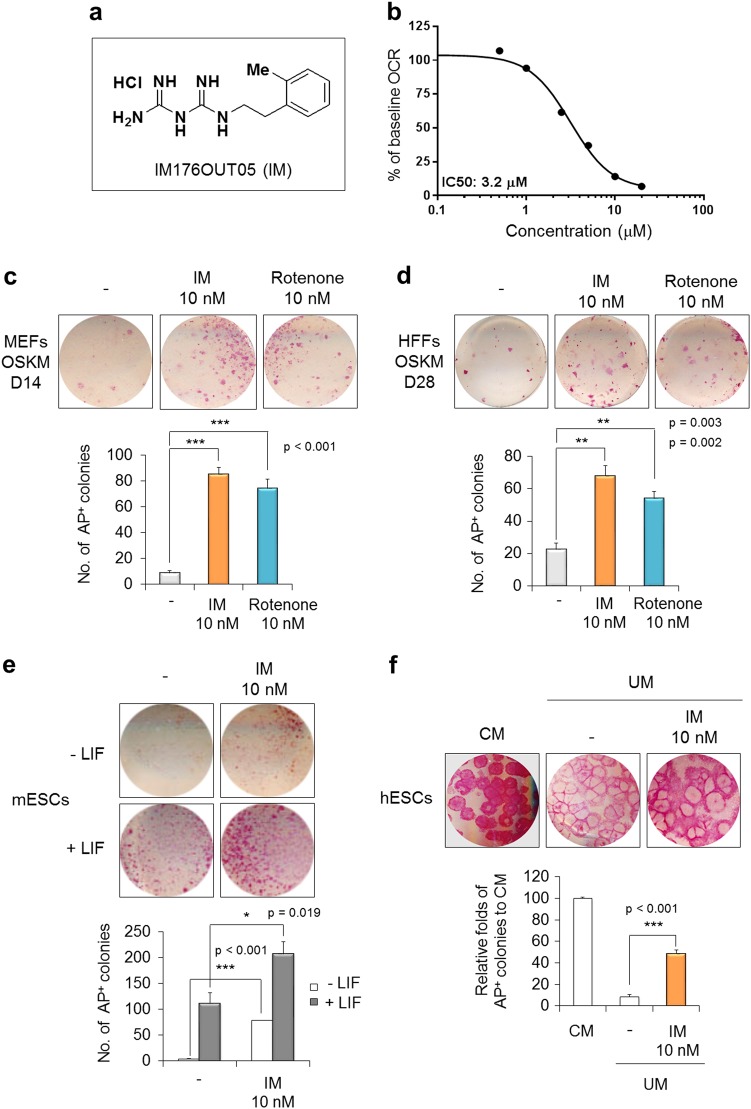
IM176OUT05 (IM) improves the acquisition and maintenance of stem cell pluripotency. **a** Chemical structure of IM. **b** Dose-response curve of the inhibition of the ETC by IM in the A549 lung carcinoma cell line. Cells were treated with serially diluted IM for 24 h, and the basal OCR was measured using a Sea Horse XF Analyzer. **c** MEFs and **d** HFFs were reprogrammed into iPSCs with OSKM reprogramming factors in the presence of 10 nM IM or rotenone. Representative images of AP^+^ colonies are shown (top). The total number of AP^+^ colonies was counted on day 14 (D14, MEFs) or day 28 (D28, HFFs) of reprogramming (bottom). **e** mESCs cultured under the non-self-renewing conditions (−LIF) or self-renewing conditions (+LIF) were treated with 10 nM IM for 4 days. Representative images of AP^+^ colonies (top) and the total number of AP^+^ colonies are shown (bottom). **f** hESCs were maintained under the self-renewing condition (CM). hESCs cultured under non-self-renewing conditions (UM) were treated with 10 nM IM for 6 days. Representative images of AP^+^ colonies (top) are shown, and the relative AP expression was quantified by scanning densitometry (bottom). *p < 0.05; **p < 0.01; ***p < 0.001 (Student’s *t*-test)

The newly synthesized novel small molecule IM (N-1-(2-methyl) phenethyl biguanide hydrochloride), which has a biguanide core, is a hydrophobic cation (Fig. 1a). IM did not show cytotoxicity in cancer cells up to 100 μM in media with a normal glucose concentration (Supplementary Figure S1a), but the cells were susceptible to IM under glucose-deprived conditions in which cells rely on mitochondrial ETC for energy generation (Supplementary Figure S1b). Therefore, IM likely inhibits mitochondrial function, and indeed, IM reduced the OCR as a surrogate of mitochondrial ETC activity with an IC50 of 3.2 μM (Fig. 1b).

As previously shown, a subtoxic dose of rotenone (an ETC complex I inhibitor) promoted efficient somatic cell reprogramming;^23^ therefore, we compared the effect of IM on iPSC generation with that of rotenone treatment (Fig. 1c). MEFs (Supplementary Figure S2a) and HFFs (Supplementary Figure S2b) were reprogrammed into iPSCs by introducing OSKM reprogramming factors. IM was applied at concentrations between 1 nM and 100 μM during the iPSC generation (Supplementary Figure S2) because IM did not show cellular toxicity in doses lower than 100 μM in MEFs (Supplementary Figure S1c) and OSKM-transduced MEFs (Supplementary Figure S1d). The reprogramming efficiency, which was determined by AP staining, was slightly increased but not significantly changed in the samples treated with the micromolar concentration range of IM (Supplementary Figure S2a and b). However, the number of AP^+^ colonies was substantially increased following treatment with IM in the nanomolar concentration range (Supplementary Figure S2a and b). IM (10 nM) and rotenone (10 nM) increased the reprogramming efficiency 9.8-fold and 8.5-fold in the MEFs (Fig. 1c) and 3.0-fold and 2.4-fold in the HFFs (Fig. 1d) over that in each untreated control, respectively. These promoting effects were also observed under conditions using three reprogramming factors without c-Myc (OSK) but were not observed under conditions without reprogramming factors, with single factors, or with OS only transduction (Supplementary Figure S2c). Additionally, the application of IM in the nanomolar concentration range was beneficial to maintain stemness in both mouse and human ESCs (Supplementary Figure S3a and b). IM (10 nM) supported the maintenance of the undifferentiated state of ESCs even under a non-self-renewing condition, namely −LIF for mESCs (Fig. 1e) and UM for hESCs (Fig. 1f). Thus, the optimal low dose of IM can improve the acquisition and maintenance of stem cell pluripotency during the generation process of both mouse and human iPSCs.

Because IM is a derivative of biguanide, we compared the effects of other known biguanides on stem cell pluripotency (Supplementary Figure S4). Metformin slightly increased the reprogramming efficiency at 10 nM as determined by AP staining and Oct4-GFP expression in OG2 (Oct4-GFP transgenic mice)-MEFs; however, metformin did not have an effect comparable to that of 10 nM IM (Supplementary Figure S4a). Phenformin, another biguanide, had no effect on the reprogramming efficiency of MEFs (Supplementary Figure S4b). Additionally, the maintenance of the stemness of mESCs (Supplementary Figure S4c) and hESCs (Supplementary Figure S4d) was slightly favorable following the application of metformin at the nanomolar concentration range, but this effect did not reach the level observed following the application of 10 nM IM.

### IM facilitates the transition of glycolytic metabolism and induction of the expression of pluripotency-related genes

**Fig. 2:**
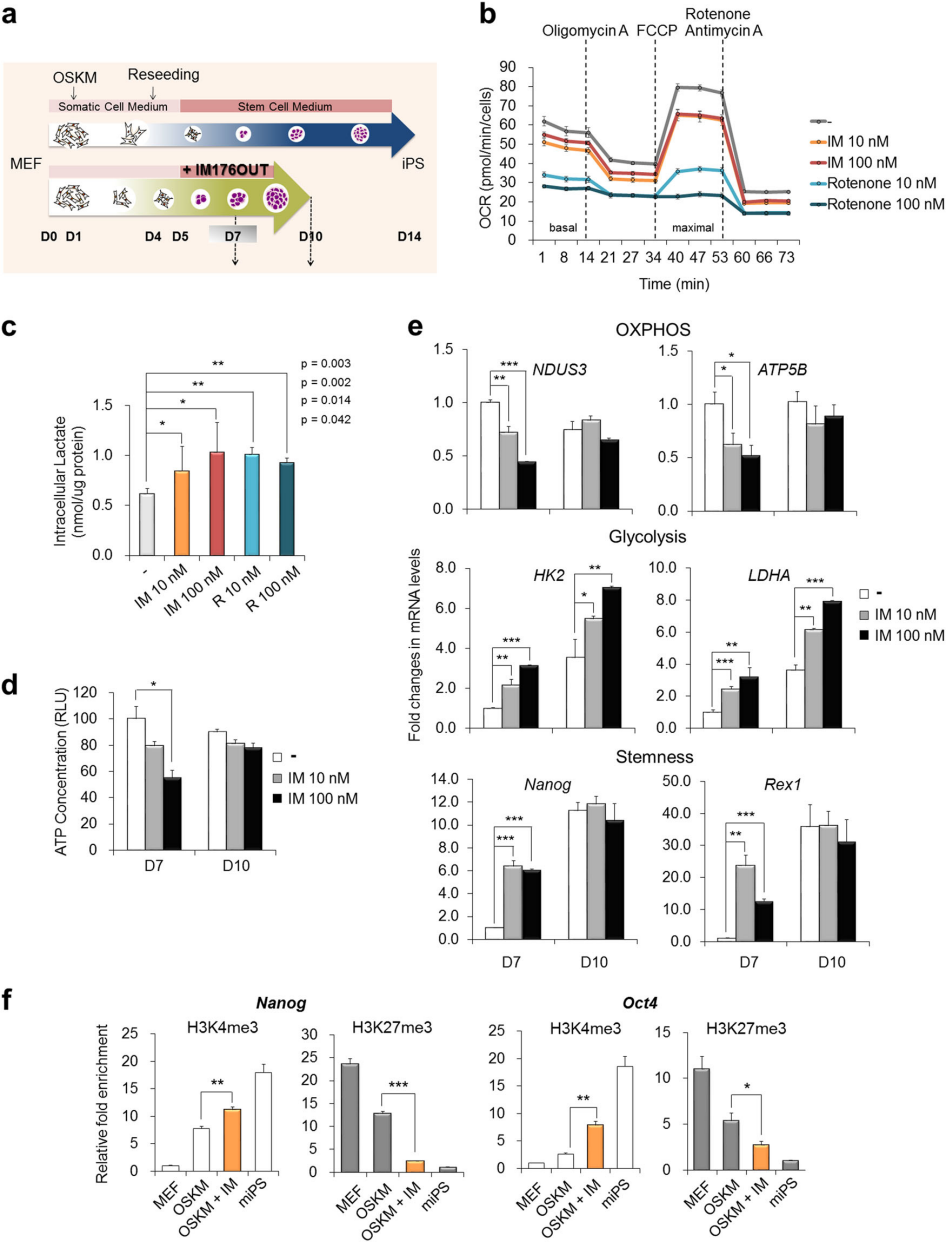
IM inhibits mitochondrial OXPHOS and increases lactate production by inducing the expression of glycolysis- and pluripotency-related genes. **a** Schematic diagram of the reprogramming process. **b** OSKM-transduced MEFs were reseeded in poly-l-lysine-coated 96-well XF plates on D4. On the following day (D5), the medium was replaced with mESC medium and each indicated chemical. After 2 days (D7), the OCR was measured using an XFe96 Flux analyzer. An ATP synthase inhibitor (1.5 μM oligomycin, ETC complex V inhibitor), uncoupler (5 μM FCCP), and complex I inhibitor (0.5 μM rotenone) + complex III inhibitor (0.5 μM antimycin A) were sequentially added at each indicated time point. **c** Lactate production was measured in each treatment group on day 7 of reprogramming. **d** The ATP concentration was quantified in each treatment group on days 7 (D7) and 10 (D10) of reprogramming. **e** The expression of the indicated gene in each treatment group was measured using real-time PCR analysis on days 7 and 10 of reprogramming. *β-Actin* expression was used as an internal control. **f** ChIP assays were performed on day 7 of reprogramming with or without IM treatment. MEFs and miPSCs were used as negative/positive controls for each histone mark. Histone H3 lysine 4 trimethylation (H3K4me3) and lysine 27 trimethylation (H3K27me3) were precipitated, and the *Nanog* and *Oct4* promoter loci were determined by real-time PCR. Input samples were used as a relative control. *p < 0.05; **p < 0.01; ***p < 0.001 (Student’s *t*-test)

Subsequently, we examined the cellular changes following IM treatment during the early stage of iPSC generation (Fig. 2a). Compared with the untreated controls, IM treatment not only increased the reprogramming efficiency but also facilitated the reprogramming kinetics (Supplementary Figure S5). Following IM treatment, colonies started to appear earlier on day 7, and the size of the colonies was larger than that in the untreated controls on days 9 and 11 (Supplementary Figure S5a). The time course required for colony selection was also shortened by approximately 10 days after transduction in the IM-treated group but was delayed over 5 days in the untreated controls (Supplementary Figure S5b). Additionally, the numbers and size of the Nanog^+^ or SSEA1^+^ fluorescent clusters were increased in the IM-treated group compared with those in the untreated controls at an early time point of reprogramming (from 7 to 9 days after transduction) (Supplementary Figure S5c).

Furthermore, we investigated the changes in bioenergetics following IM treatment during the early stage of reprogramming. The basal OCRs (before inhibitor treatment) were slightly inhibited by treatment with 10 nM or 100 nM IM compared with those in the DMSO controls on day 7 of reprogramming, and the maximal OCRs were considerably inhibited by IM treatment (Fig. 2b). Rotenone potently reduced both the basal and maximal OCR in a dose-dependent manner (Fig. 2b). The basal ECAR was inversely correlated with the OCR, and IM and rotenone treatment clearly increased the acidification of the medium, revealing increased glycolytic activity (Supplementary Figure S5d). The intracellular production of lactate directly shows that, compared with the untreated controls on day 7, the end-product of glycolysis was increased in the cells treated with 10 nM and 100 nM IM or rotenone (Fig. 2c). The cellular ATP content was reduced by IM treatment on day 7 as a decrease in the energy-generating mitochondrial function accompanying OCR reduction (Fig. 2d). However, the ATP content was recovered on day 10 due to compensation by increased glycolysis, which is an alternative energy-generating pathway that overcomes energy deficits, in the IM-treated cells (Fig. 2d). Therefore, these results show that 10 nM IM slightly interfered with oxygen consumption but was sufficient to accelerate the glycolytic metabolic transition. The changes in gene expression were correspondently observed; compared with each control, the expression levels of *NDUS3* (an ETC complex I enzyme) and *ATP5B* (an ETC complex V enzyme) were downregulated in the IM-treated cells on day 7 but were recovered on day 10 (Fig. 2e). By contrast, the expression levels of *HK2* and *LDHA* (major enzymes of glycolysis) were upregulated in IM-treated cells on day 7, and further induction was found during the progression of reprogramming on day 10. Notably, the expression levels of *Nanog* and *Rex1* (pluripotency-related genes) were potently upregulated in IM-treated cells during the early stages of reprogramming and was saturated in all groups on day 10. More evidently, the occupancies of the active histone mark (H3K4me3) and repressive histone mark (H3K27me3 and H3K9me2) were enriched and decreased, respectively, at the *Nanog* and *Oct4* loci following IM treatment on day 7 (Fig. 2f and Supplementary Figure S5e). These observations suggest that IM can promote glycolytic metabolic reprogramming and pluripotency induction during the early stage of the cellular reprogramming process. Thus, we explored the effect of the application of IM on tissue regeneration, specifically hair follicle regrowth.

### IM promotes hair regrowth in mice

**Fig. 3:**
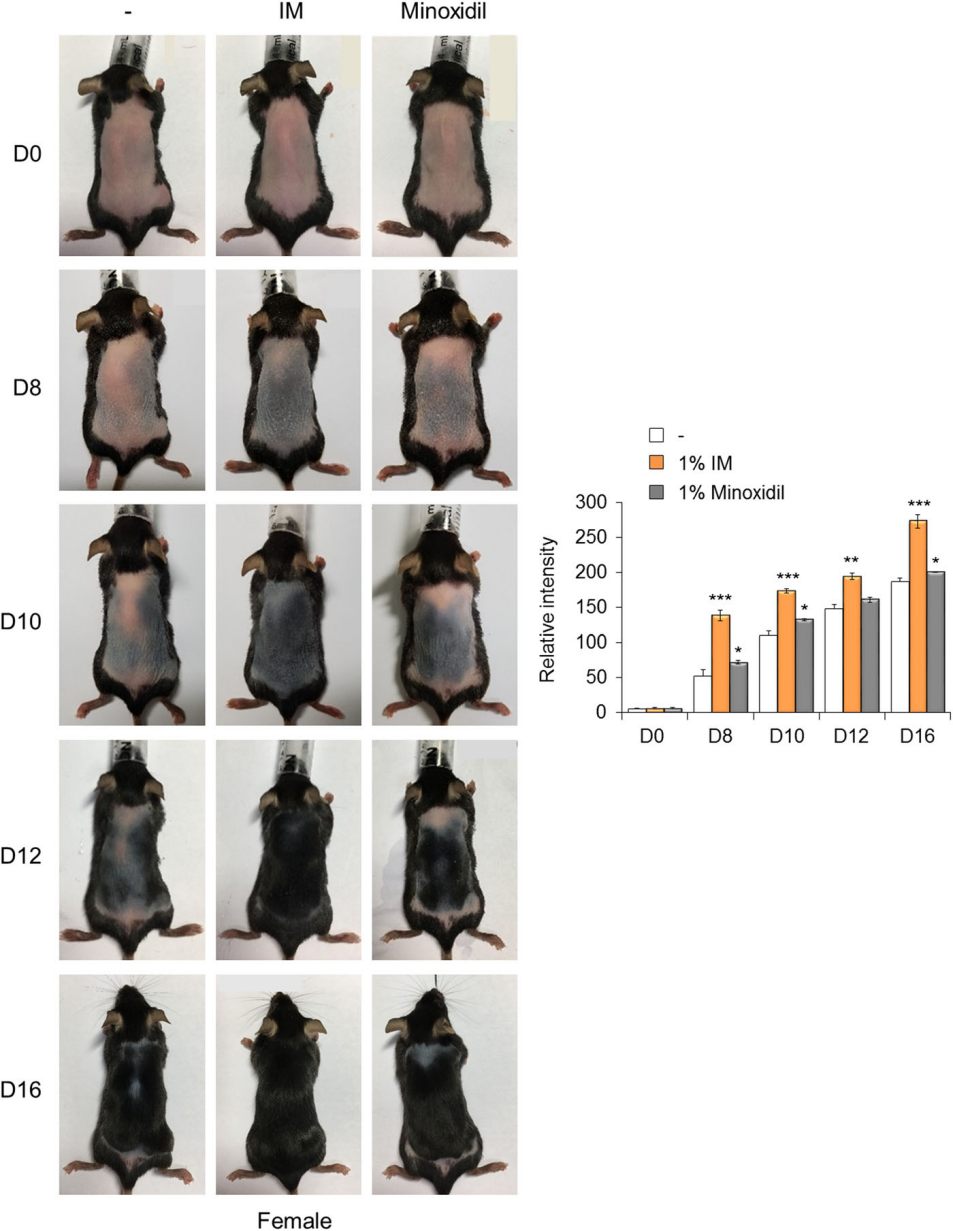
IM promotes hair regrowth in mice. C57BL/6 mice in the telogen phase (7 weeks old) were depilated. Placebo control (−), 1% IM, or 1% minoxidil was topically applied daily to the dorsal skin. Representative photos of mice showing skin color darkness and hair regrowth on days 8, 10, 12, and 16 (left). The level of pigmentation was quantified by the intensity of the darkness of the back skin in the same area (right). *p < 0.05; **p < 0.01; ***p < 0.001 (Student’s *t*-test)

Preliminarily, we tested whether IM could enhance hair regrowth in mice without toxicity or other side effects (Supplementary Figure S6). The hair cycle was synchronized by the depilation of telogen phase hairs from 7-week-old C57BL/6 mice^1^, and various concentrations of IM were topically applied daily to the dorsal skin of the mice (Supplementary Figure S6). On day 9, dramatic changes were observed in the area treated with 1% IM, and black pigmentation and hair growth were robustly detected. Hair regrowth was not observed in the control areas or areas treated with 0%, 0.1%, or 0.5% IM, although pigmentation developed on day 11. Next, we compared the abilities of IM and minoxidil, which is an approved drug to treat hair loss, to promote hair regrowth in both male and female mice. IM treatment had a strong promoting effect on hair regrowth, especially in female mice (Fig. 3). By day 8, the skin color was clearly distinguished with darkening in IM-treated mice compared with that in either the control or minoxidil-treated mice. By day 10, hair regrowth was distinctly promoted by IM treatment (Fig. 3) and was reproducibly observed in an independent group of female mice (Supplementary Figure S7). These phenomena were similarly observed in the male mice, but the effect of IM was comparable to that of minoxidil in the male mice (Supplementary Figure S8). Rashes and scars were detected in a few mice after depilation (Supplementary Figure S8b, D0); however, adverse effects, such as skin problems induced by IM treatment, were not observed in any of the animals in the in vivo experiments.

### IM facilitates the cycle of hair follicle regeneration in mice

**Fig. 4:**
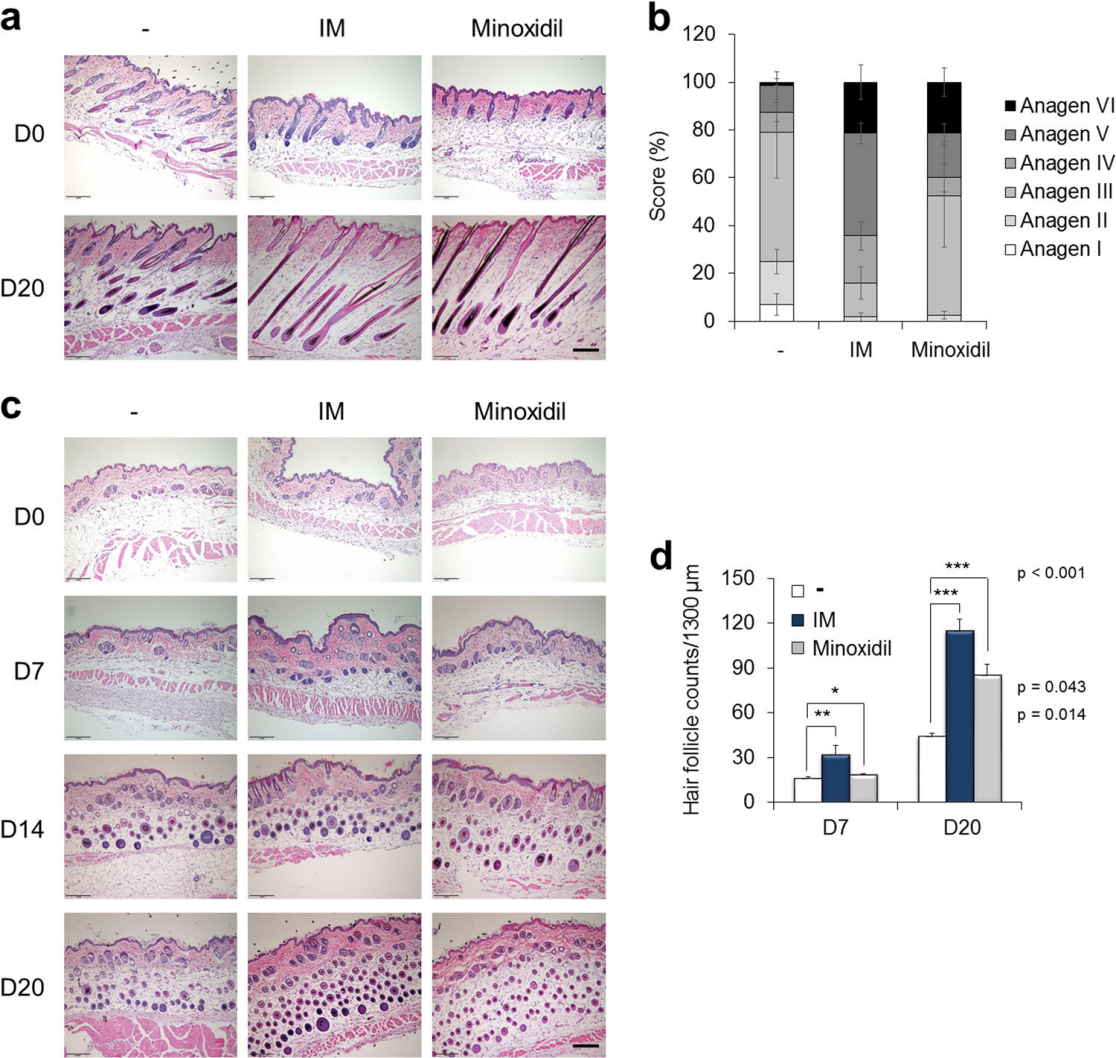
IM facilitates the cycle of hair follicle regeneration in mice. **a** Representative images of H&E-stained longitudinal sections of each treated mouse skin on days 0 and 20 after depilation. **b** Progression of the hair follicle cycle on day 20 was quantitatively evaluated. Individual hair follicles were classified based on the classification by Chase. **c** Representative images of H&E-stained transverse sections of each treated mouse skin on days 0, 7, 14, and 20 after depilation. **d** Hair follicles were counted on days 7 and 20. *p < 0.05; **p < 0.01; ***p < 0.001 (Student’s *t*-test). Scale bar = 200 μm

**Fig. 5:**
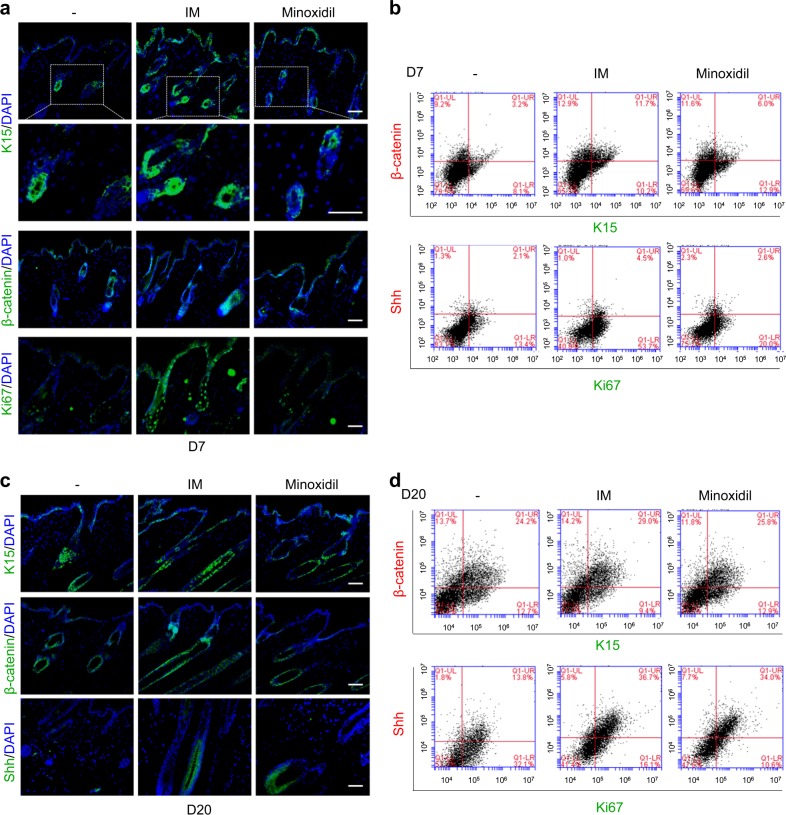
IM promotes the expression of hair follicle cycling-related markers. **a** Immunohistochemistry of K15, β-catenin, and Ki67 in each treated mouse skin on day 7. Enlarged images of K15 in the top panels (dotted line) are shown in the second panels. **b** FACS analysis of K15/β-catenin and Ki67/Shh in single cells of each treated mouse skin on day 7. **c** Immunohistochemistry of K15, β-catenin, and Shh in each treated mouse skin on day 20. **d** FACS analysis of K15/β-catenin and Ki67/Shh in single cells of each treated mouse skin on day 20. DAPI staining was used to identify the nuclei (blue). Scale bar = 50 μm

The tissues were histomorphometrically analyzed based on the classification by Chase^1,30^ to show that IM stimulated the progression of the hair follicle cycle (Fig. 4a). On day 20, most hair follicles in the control mice were quantitatively in the anagen III stage, but most hair follicles in the IM-treated mice were at a later stage of anagen, mainly anagen stages V and VI, in longitudinal sections (Fig. 4b). Additionally, the number of hair follicles was obviously increased in the IM-treated mice compared with that in the control mice in transverse sections on day 7 (Fig. 4c), and the number in the IM-treated or minoxidil-treated mice was greater than that in the control mice on day 20 (Fig. 4d). Moreover, keratin 15 (K15), which is a marker of hair follicle stem cells^31^, was strongly expressed in the hair follicle bulge area of the IM-treated mice compared with that in either the control or minoxidil-treated mice on day 7 (Fig. 5a). The expression of β-catenin, which mediates hair follicle regeneration^32^, was also increased in the IM-treated mice on day 7. At this time point, Ki67-positive proliferating cells were already apparent in the IM-treated mice, indicating that hair follicle cycling and further expansion of proliferating progenitors were augmented by IM (Fig. 5a). The K15^+^/β-catenin^+^ populations were quantified by FACS analysis, and these populations occupied 3.2%, 11.7%, and 6.0% of single cells in the skin of the control and IM-treated, and minoxidil-treated mice, respectively (Fig. 5b). A 3.7-fold increase was observed in the IM-treated mice over the control mice (Fig. 5b). By day 20, K15 and β-catenin were strongly detected in all groups of mice (Fig. 5c), and the K15^+^/β-catenin^+^ populations were represented at over 24% in all groups (Fig. 5d). Shh, which is another essential factor for hair follicle development^33^, was clearly detected in the IM-treated or minoxidil-treated mice (Fig. 5c). The Ki67^+^/Shh^+^ populations accounted for <5% in all groups of mice on day 7 (Fig. 5b), but this percentage was significantly increased on day 20 by 13.8%, 36.7%, and 34% in the single cells in the skin of the control, IM-treated, or minoxidil-treated mice, respectively (Fig. 5d). On day 20, the Ki67^+^/Shh^+^ populations were increased by 2.7-fold in the IM-treated mice over those in the control mice (Fig. 5d).

### IM activates stem cell metabolism and further expansion of proliferating progenitors during the early phases of hair follicle regeneration

**Fig. 6:**
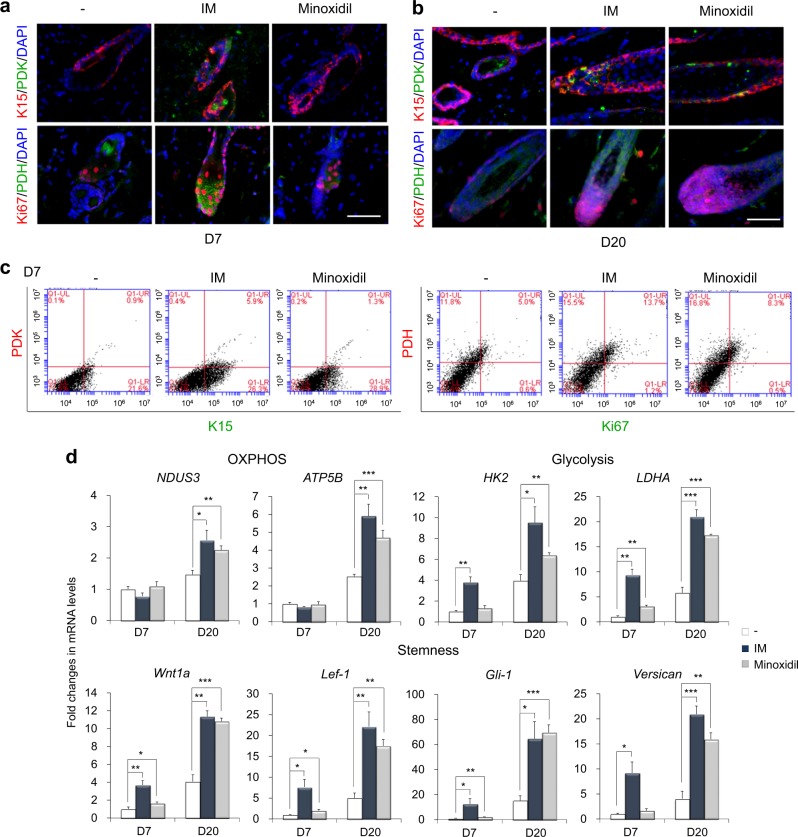
IM stimulates the expression of stem cell metabolism-related and hair follicle regeneration-related genes during the early phases of hair regrowth. Immunohistochemistry of K15, PDK, Ki67, and PDH in each treated mouse skin on **a** day 7 and **b** day 20. DAPI staining was used to identify the nuclei (blue). Scale bar = 50 μm. **c** FACS analysis of K15/PDK and Ki67/PDH in single cells of each treated mouse skin on day 7. **d** Expression of the indicated gene in each treated mouse was quantified by real-time PCR analysis on days 7 and 20 after depilation. *β-Actin* expression was used as an internal control. *p < 0.05; **p < 0.01; ***p < 0.001 (Student’s *t*-test)

A recent report provided data showing that K15-positive hair follicle stem cells highly express pyruvate dehydrogenase kinase (PDK), which is an enzyme that promotes glycolytic conversion^34^, whereas Ki67-positive proliferating cells strongly express pyruvate dehydrogenase (PDH), which is an enzyme responsive to mitochondrial OXPHOS^12^. PDK expression was mainly detected in the K15-positive stem cells in the IM-treated mice but was not detected in either the control or minoxidil-treated mice on day 7 (Fig. 6a). The K15^+^/PDK^+^ populations were hardly detected in the control or minoxidil-treated mice but occupied 5.9% of the single cells in the IM-treated mice skin by FACS analysis on day 7 (Fig. 6c). These results showed that the IM treatment increased the glycolytic stem cell population (K15^+^/PDK^+^) by 6.6-fold over that in the control mice. PDH expression was obvious in the Ki67-positive proliferating cells in the IM-treated mice on day 7 (Fig. 6a) and was detected in the highly proliferative cells in all groups on day 20 (Fig. 6b). The Ki67^+^/PDH^+^ populations were also increased in the IM-treated mice by 13.7%, whereas these populations represented 5.0% and 8.3% of cells, respectively, in the control and minoxidil-treated mice on day 7 (Fig. 6c). Subsequently, we examined the reciprocal expression patterns of each marker, and the discrete expression of K15/PDH and Ki67/PDK was obviously detected in the IM-treated mice on day 20 (Supplementary Figure S9a), clearly distinguishing the PDK-expressing glycolytic K15-positive hair follicle stem cells from PDH-expressing OXPHOS-dependent Ki67-positive proliferating cells. Moreover, FACS analysis apparently revealed that the populations of PDK^+^ and Ki67^+^ cells were well separated and that each population was increased by the IM or minoxidil treatment on day 20 (Supplementary Figure S9b). Separated populations of PDH^+^ and K15^+^ cells were also detected and increased by IM or minoxidil treatment on day 20 (Supplementary Figure S9b).

Finally, the expression of genes related to metabolism and hair regeneration was analyzed in skin tissues from each treated mouse (Fig. 6d). The expression of glycolysis-related enzymes (*HK2* and *LDHA*) was prominently induced in the IM-treated mice by day 7 and was significantly accelerated in both the IM-treated and minoxidil-treated mice compared with that in the control mice on day 20. At this stage, the expression of OXPHOS-related enzymes (*NDUS3* and *ATP5B*) was elevated in both the IM- and minoxidil-treated mice compared with that in the control mice, implying that IM facilitates glycolytic metabolic transition during the early phases of hair follicle regeneration, resulting in the further promotion of OXPHOS during the later phase and contributing to the active energy metabolism required for hair regrowth. Importantly, the expression of hair follicle development-related genes (*Wnt1a*, *Lef-1*, *Gli-1*, and *Versican*) was potently upregulated in the IM-treated mouse skin by day 7, and minoxidil also slightly increased the expression of *Wnt1a*, *Lef-1*, and *Gli-1* compared with that in the control. By day 20, compared with control mice, both the IM-treated and minoxidil-treated mice showed pronounced induction of stemness-related genes (Fig. 6d). Overall, IM promotes an increase in the glycolytic hair follicle stem cell population during the early phases of tissue regeneration, possibly conferring advantages in the progression to the next stages of the regeneration process.

We also compared the effect of a biguanide, metformin, with that of IM on hair regrowth (Supplementary Figure S10). The intensity of the darkness of the back skin was slightly increased in the metformin-treated mice compared with that in the control mice by day 8, but the effects were not significantly, consistent with the in vitro data (Supplementary Figure S4). Additionally, the effect of IM on tissue regeneration was observed in pinnal tissue repair (Supplementary Figure S11). IM dose dependently decreased the wound area in an ear hole punch assay. Thus, IM could possibly activate tissue regeneration other than hair regrowth.

## Discussion

More fundamental therapies for alopecia (hair loss) are needed in addition to surgical management, and emerging studies are focusing on hair follicle regeneration, particularly stem cell regulation. Here, we provide a novel and safe small molecule, IM, which promotes the acquisition and maintenance of stemness in both pluripotent stem cells and adult tissue stem cells with a glycolytic metabotype. IM increased glycolytic metabolism in stem cells by mildly inhibiting mitochondrial OXPHOS, which could facilitate the cycle of hair follicle regeneration by increasing stem cell populations. In particular, because IM accumulates in mitochondria via the mitochondrial membrane potential, IM accrues at higher levels in stem cells with a higher mitochondrial membrane potential^19^, possibly offering functional selectivity toward stem cells.

The hair follicle is the most proliferative tissue in the body and undergoes repeated cycles of stem cell self-renewal and differentiation throughout life. In addition to hair follicle stem cells, several other stem cells such as ESCs, NSCs, and satellite cells, prefer glycolytic cellular metabolism because glycolysis can provide conditions favorable to stem cells by supplying the building blocks (e.g., nucleotides and lipids) needed for cell proliferation and simultaneously protecting against oxidative damage from mitochondrial OXPHOS^29^. Next, the more differentiated cells primarily depend on mitochondrial respiration, which is advantageous for energy production^12^. Thus, IM can stimulate the early phase of hair follicle regeneration by providing a metabolic environment favorable to stem cells, further promoting the next stages of progression of the hair follicle cycle.

IM has a high pKa value (pKa = 12.14 ± 0.10), resulting in a positive charge at a neutral pH and low partition coefficients (logP = −0.183 ± 0.0200), implying that IM is a low lipophilic compound. However, the ability of IM to cross cell membranes was not limited (Supplementary Figure S12). We measured the penetrated drug concentration in various cell lines with different levels of organic cation transporter 1 (OCT1) expression (SK-OV-3, MDA-MB-231 > MDA-MB-435 > 786-O, MCF-7). The concentration of IM detected inside the cells was higher than that of metformin and was not limited by OCT1 expression (Supplementary Figure S12a), although the drug concentration following topical application was not measured. Metformin has the limitation of cell permeability and can enter cells only when the cells express a transporter that can carry a positively charged molecule, such as OCT1^35^. Due to its improved cell penetration, IM effectively activates AMPK at a much lower concentration (EC50 = ~5 μM) (Supplementary Figure S12b) compared with metformin (EC50 = 1 mM) (Supplementary Figure S12c) in MCF-7 cells. The OCR was also inhibited at a substantially lower concentration of IM (IC50 = 3.2 μM) (Fig. 1b) compared with that of metformin (IC50 = 822 μM) (data not shown) in A549 cells, suggesting that IM has a 260-fold improved potency over metformin.

Biguanide metformin, which is a prescription drug for type 2 diabetes and metabolic syndrome, has received attention because of its possible repurposing functions in various disease settings, including cancer, cardiovascular diseases, and aging^36^. Metformin, which is similar to IM, has been described to exert a direct inhibitory effect on mitochondrial respiration through interference with complex I activity of the ETC, resulting in the conversion of bioenergetics to glycolysis^37^. Uncoupled respiration has also been reported to reduce the cellular energy status and subsequently activate AMPK, which can conversely stimulate Warburg-like glycolysis and then promote muscle regeneration through the activation of myogenic stem cells (satellite cells)^38^. Therefore, we expect metformin to have an effect on pluripotent stem cells and hair follicle stem cells. However, metformin at the nanomolar concentration range only slightly increased the somatic cell reprogramming efficiencies and was marginally favorable for the maintenance of stemness in ESCs (Supplementary Figure S4). The effect of metformin did not reach the level of the effect of IM, and there was no effect on hair regrowth in the mouse model (Supplementary Figure S10), which may be due to the lower potency of metformin, which has lower penetration, AMPK activity, and OCR inhibition properties than IM.

Understanding and controlling stem cell metabolism has yielded promising strategies to enhance the tissue regenerative capacity^39^. It is important to support stem cell functions that repopulate niche cells to sustain tissue regrowth in distinct cellular compartments, niches, and metabolic milieus^40,41^. For instance, intestinal niche Paneth cells provide lactate to intestinal stem cells, which fuel mitochondrial OXPHOS in stem cells and then promote their cellular differentiation^42^. K15-positive hair follicle stem cells also present glycolytic bioenergetics, whereas differentiated Ki67-positive proliferating cells mainly depend on OXPHOS with a mature mitochondrial phenotype^12^. In our study, K15-positive stem cells were strongly detected in the hair follicle bulge area of the IM-treated mice at an early time point of hair regrowth (Fig. 5a). Furthermore, PDK, which decreases pyruvate oxidation and leads to the conversion of pyruvate to lactate, was clearly detected in the K15-positive stem cells in the IM-treated mice on day 7, whereas its expression was not detected in either the control or minoxidil-treated mice at this time point (Figs. 6a, c). In addition, in contrast to minoxidil, which is a vasodilator that opens mitochondrial K^+^ ATP channels, IM prominently induced the expression of glycolysis-related enzymes (*HK2* and *LDHA*) by day 7 (Fig. 6d). Therefore, we suggest that IM can promote stem cell functions by directly supporting the metabolic preferences of stem cells and may secondarily regulate redox homeostasis.

Epithelial–mesenchymal crosstalk is essential to initiate hair follicle regeneration^3^, and epithelial Wnt/β-catenin signaling is the first key pathway in hair follicle morphogenesis^32^. Mice deficient in β-catenin or Lef1, which is an essential regulatory factor in Wnt signaling, exhibit few or no hair follicles^3^, and the Wnt1a-conditioned stem cell medium accelerates hair regrowth^31^. In our study, IM robustly induced β-catenin expression early during the regeneration phase (Fig. 5a). Compared with control mice, β-catenin expression was 1.98-fold higher in the IM-treated mice (Fig. 5b). Additionally, a major mesenchymal compartment, the dermal papilla, responds to Wnt signaling and induces hair cycling and regeneration^31^. Shh and its downstream target, Gli1 signaling, engage in crosstalk with Versican expression in dermal papilla cells, which play roles in replenishing the hair follicle niche^3^. IM promotes the expression of these hair follicle development-related genes and activates multiple instructive signals for hair regeneration both in epithelial and mesenchymal compartments (Fig. 6d).

Here, we report a novel small molecule, IM, which augments hair follicle regeneration by supporting stem cell metabolism without toxicity. IM did not appear to have any side effects by histomorphometric observation after topical application to the surface of the skin. The approved concentration of minoxidil is 5% and 2% in men and women, respectively. In our study, 1% IM was more effective than 1% minoxidil in promoting hair regrowth in female mice (Fig. 3 and Supplementary Figures S7), whereas 1% IM and 1% minoxidil both exhibited similar growth-promoting effects in male mice (Supplementary Figures S8). An analysis of the different responses to IM between female and male mice may provide therapeutic options because of the current lack of treatment for female hair loss. Further precise and continued investigations are needed to determine whether IM can repair damaged hair follicles after prolonged hair loss and whether it can also protect against further damage.

## Electronic supplementary material

Supplementary figures

Supplementary Figure Legends & Tables
