# Supplementary material for: A novel and safe small molecule enhances hair follicle regeneration by facilitating metabolic reprogramming
Source: Exp Mol Med. 2018 Dec 6;50(12):1–15. doi: 10.1038/s12276-018-0185-z (PMC6283868; doi:10.1038/s12276-018-0185-z)

Supplementary Figure 1

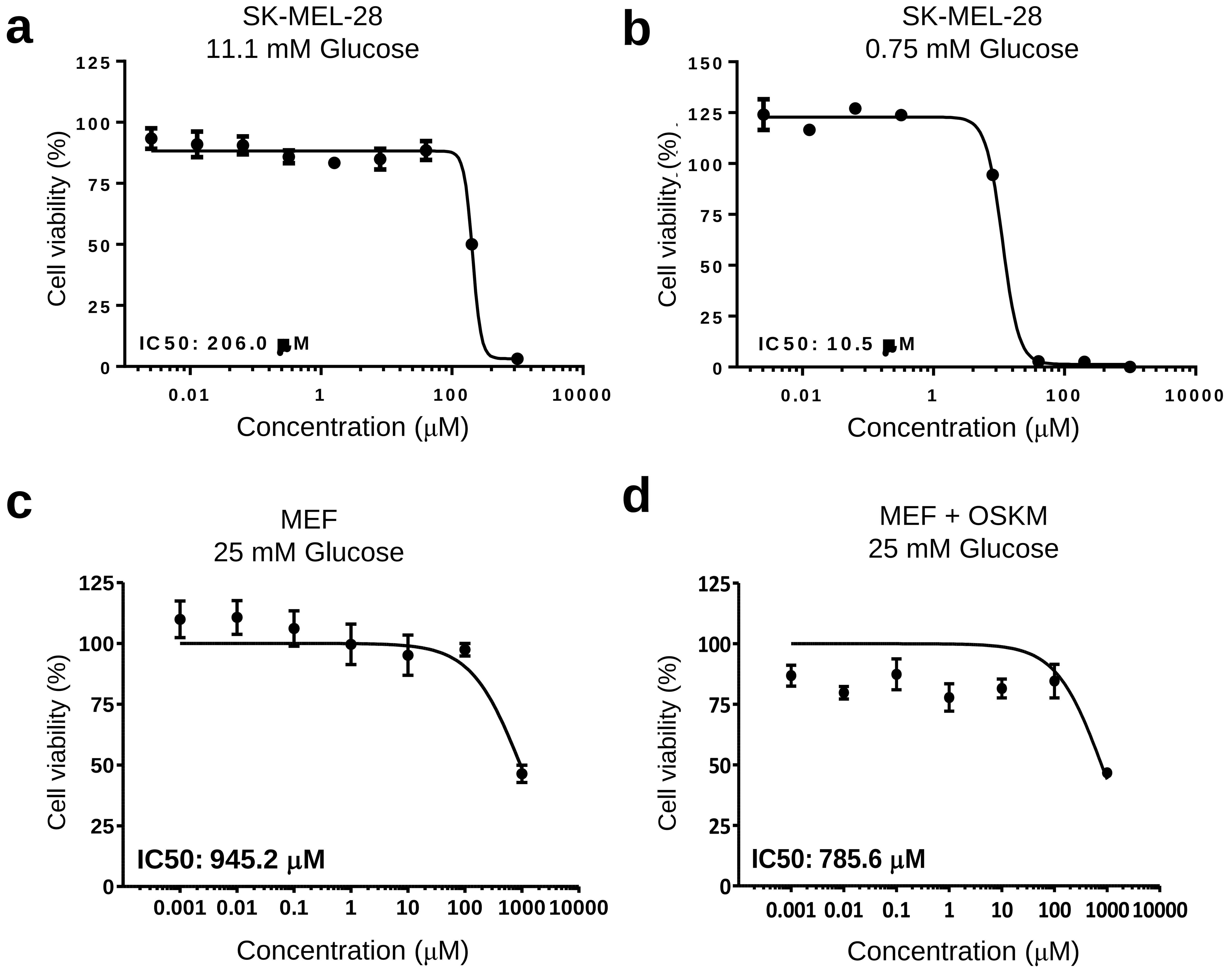

Supplementary Figure 2

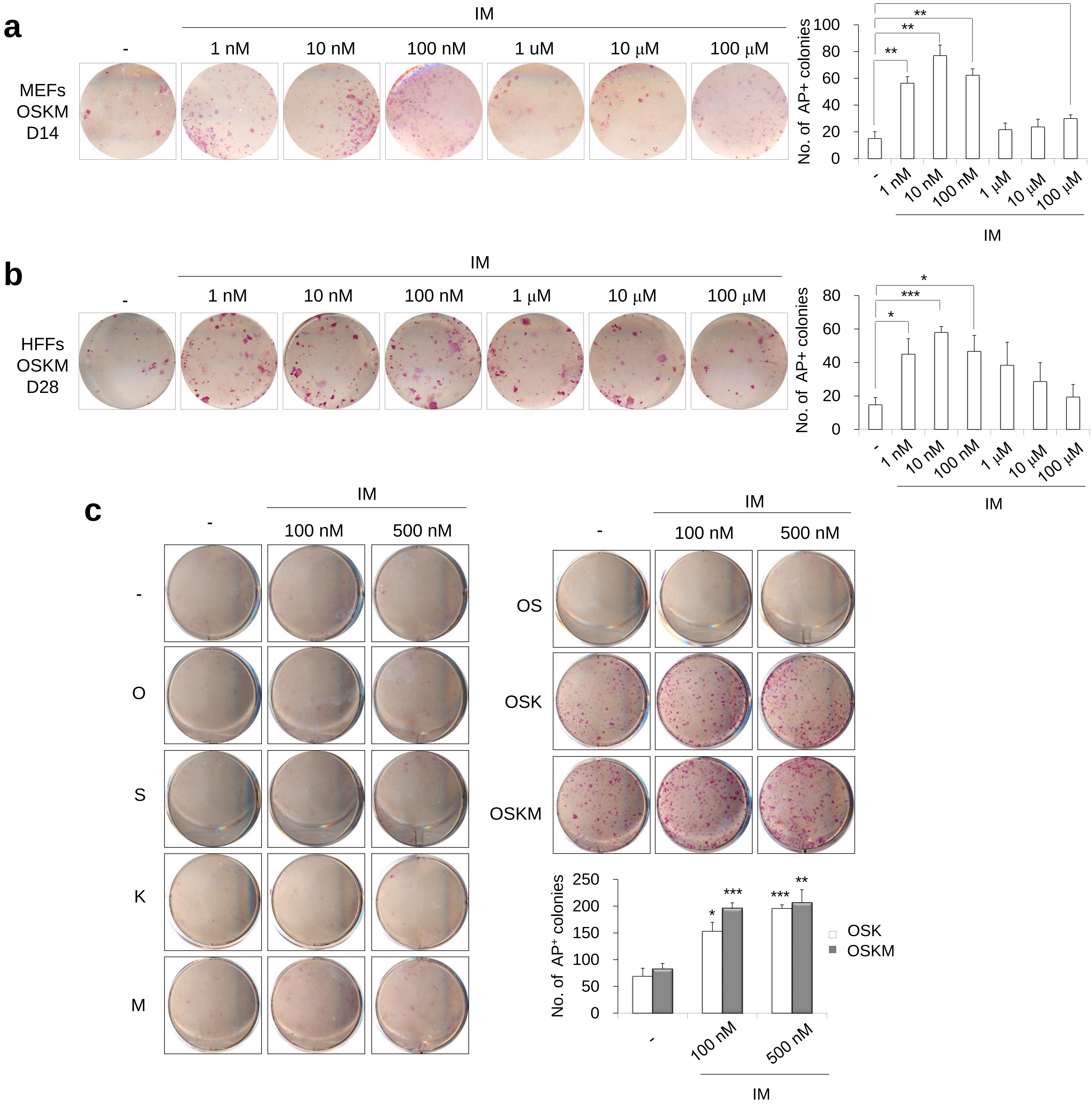

Supplementary Figure 3

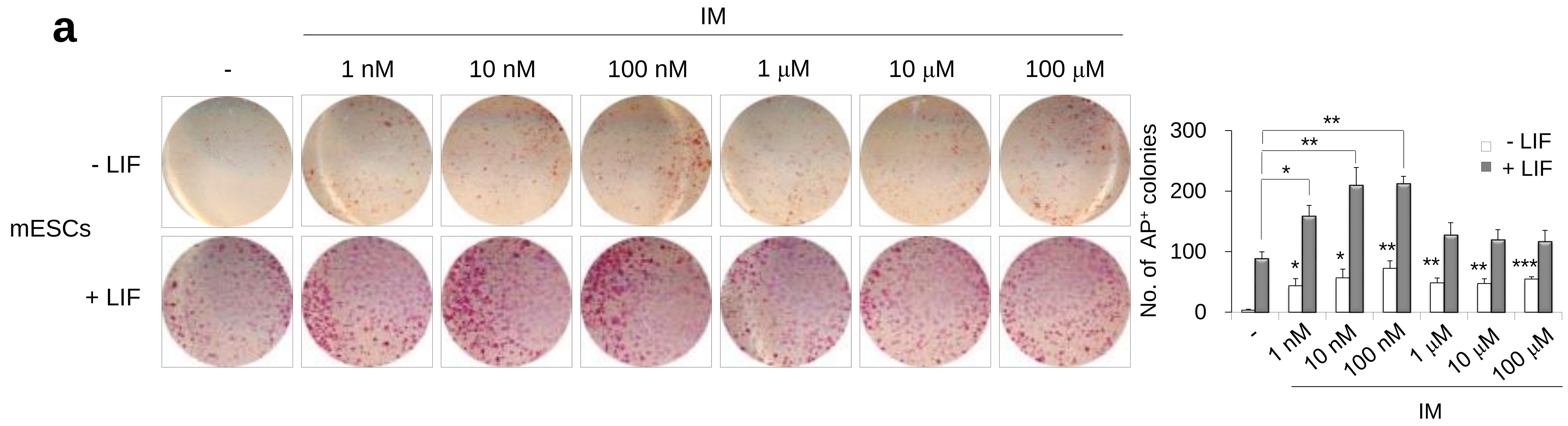

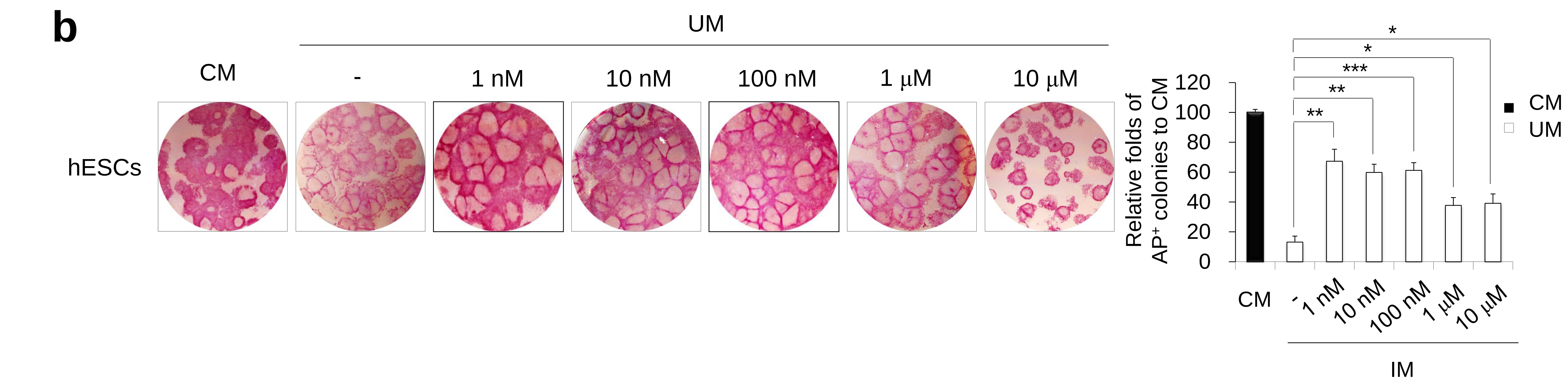

Supplementary Figure 4

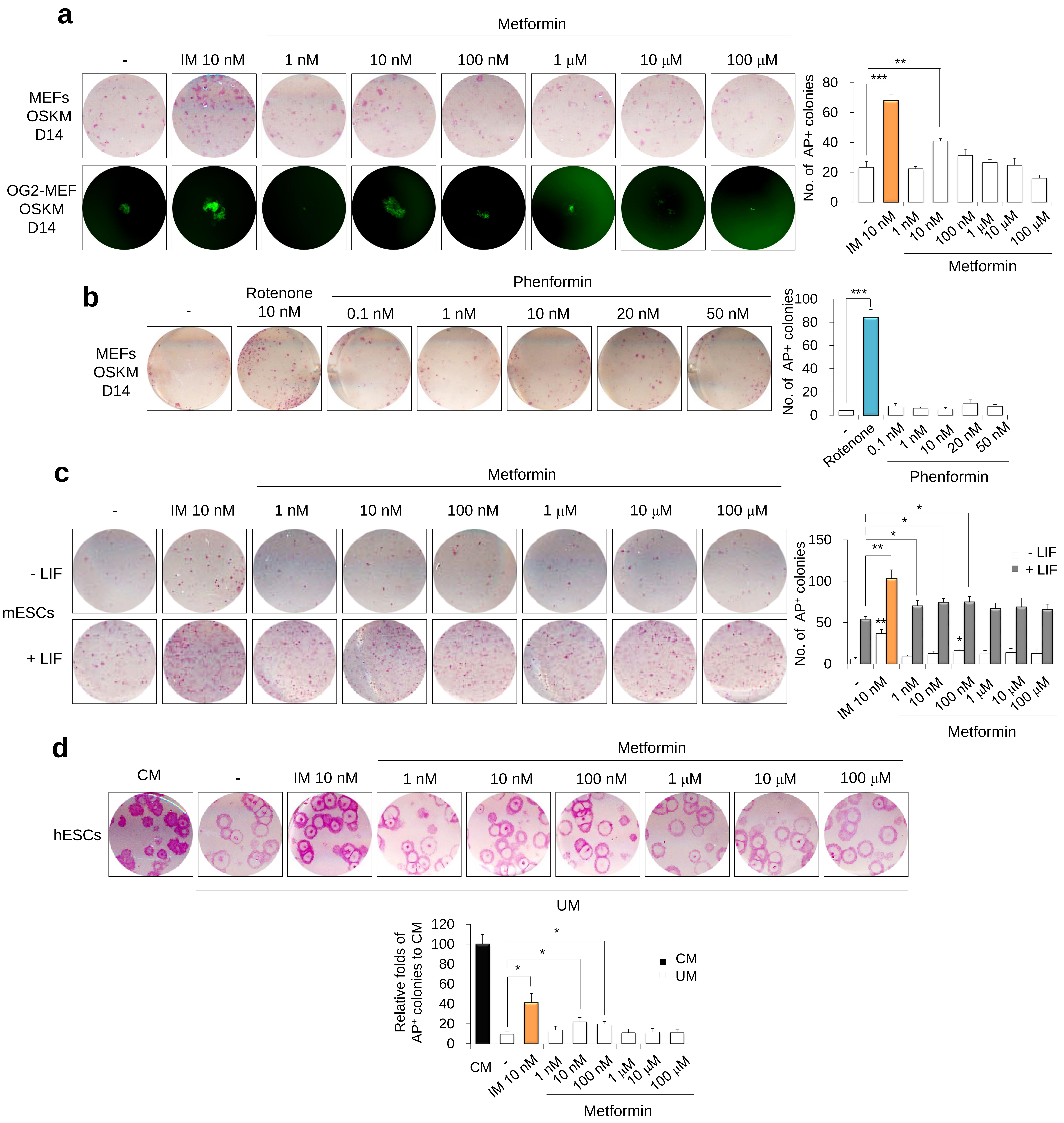

Supplementary Figure 5

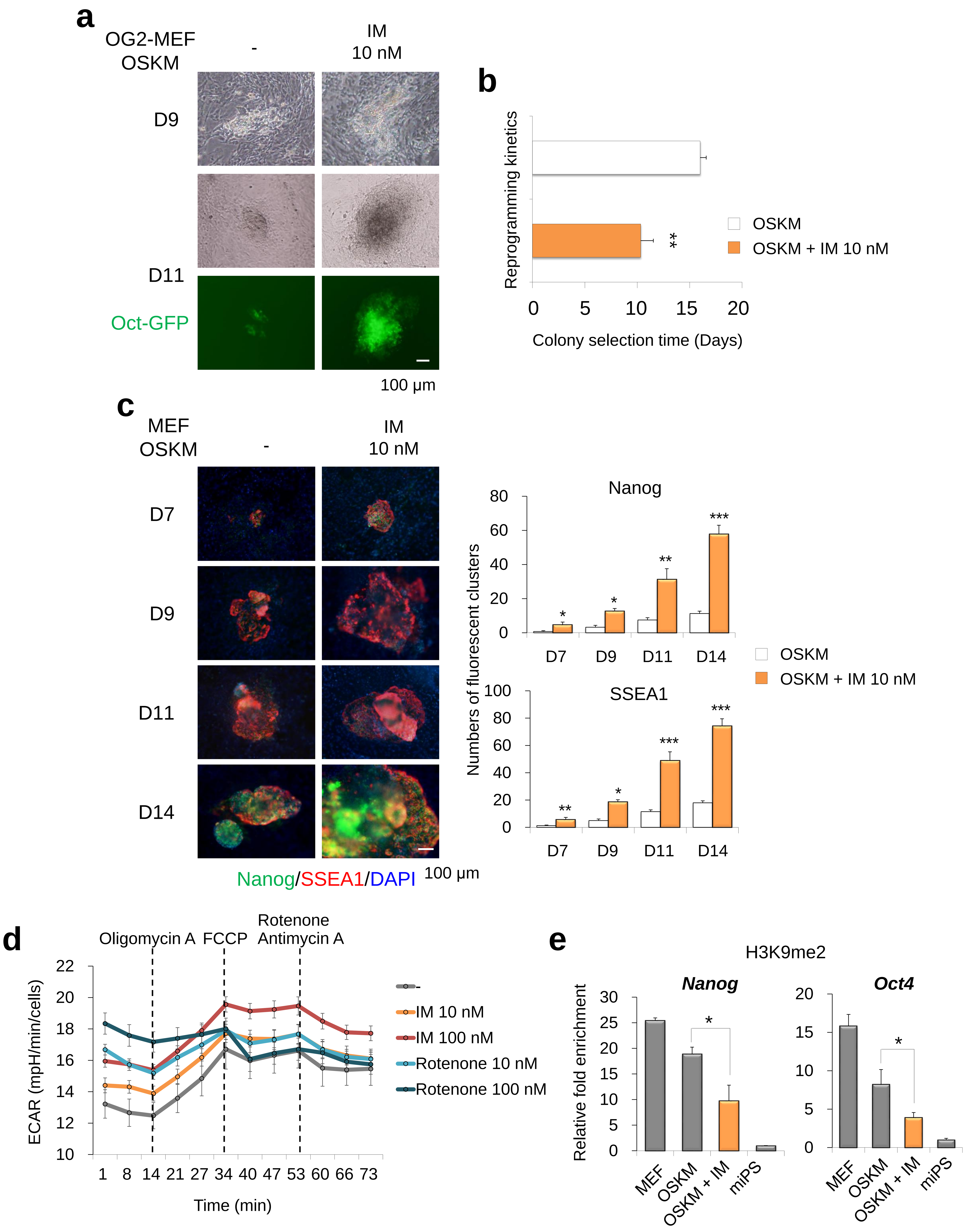

Supplementary Figure 6

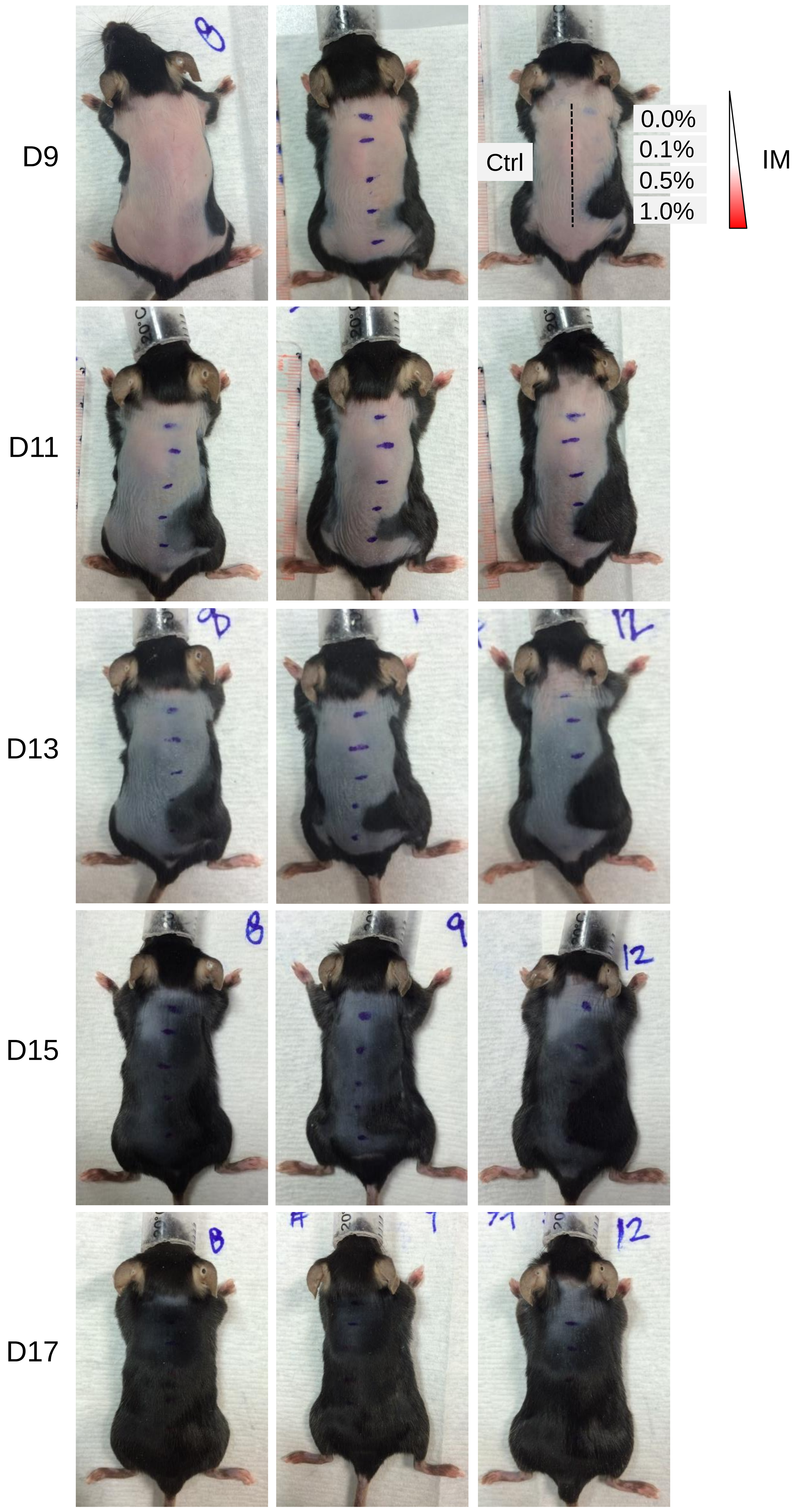

Supplementary Figure 7

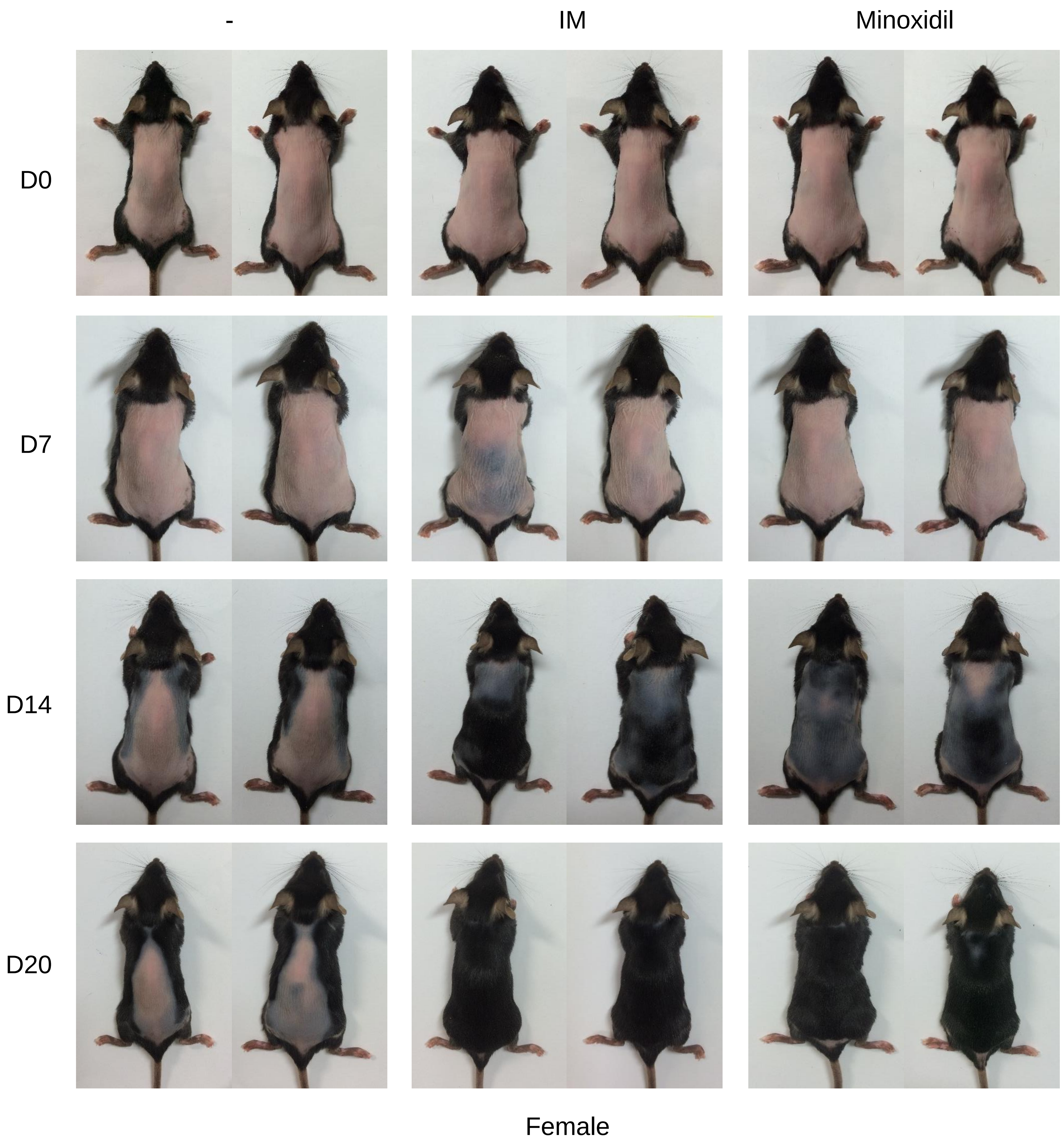

Supplementary Figure 8

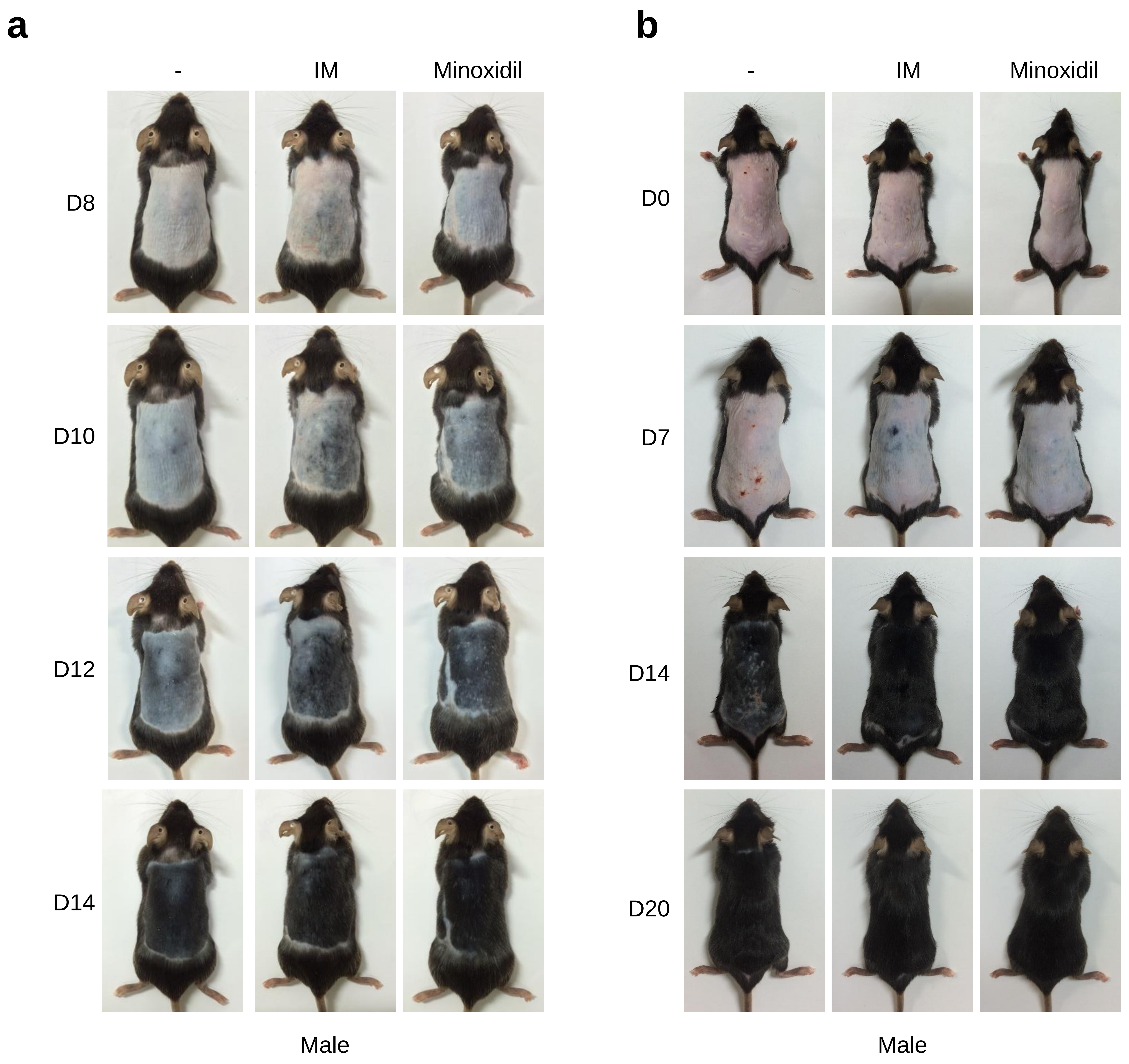

Supplementary Figure 9

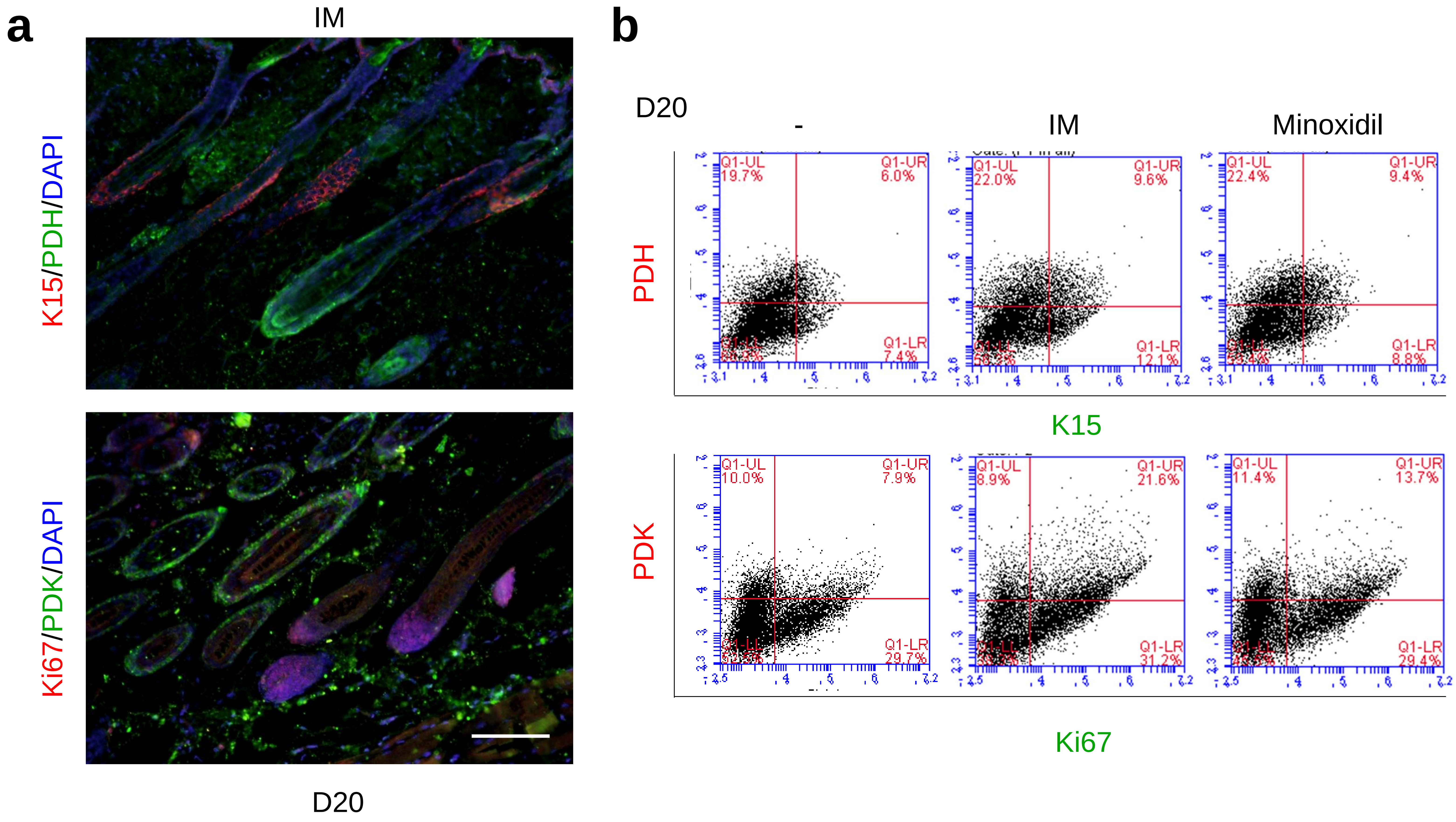

Supplementary Figure 10

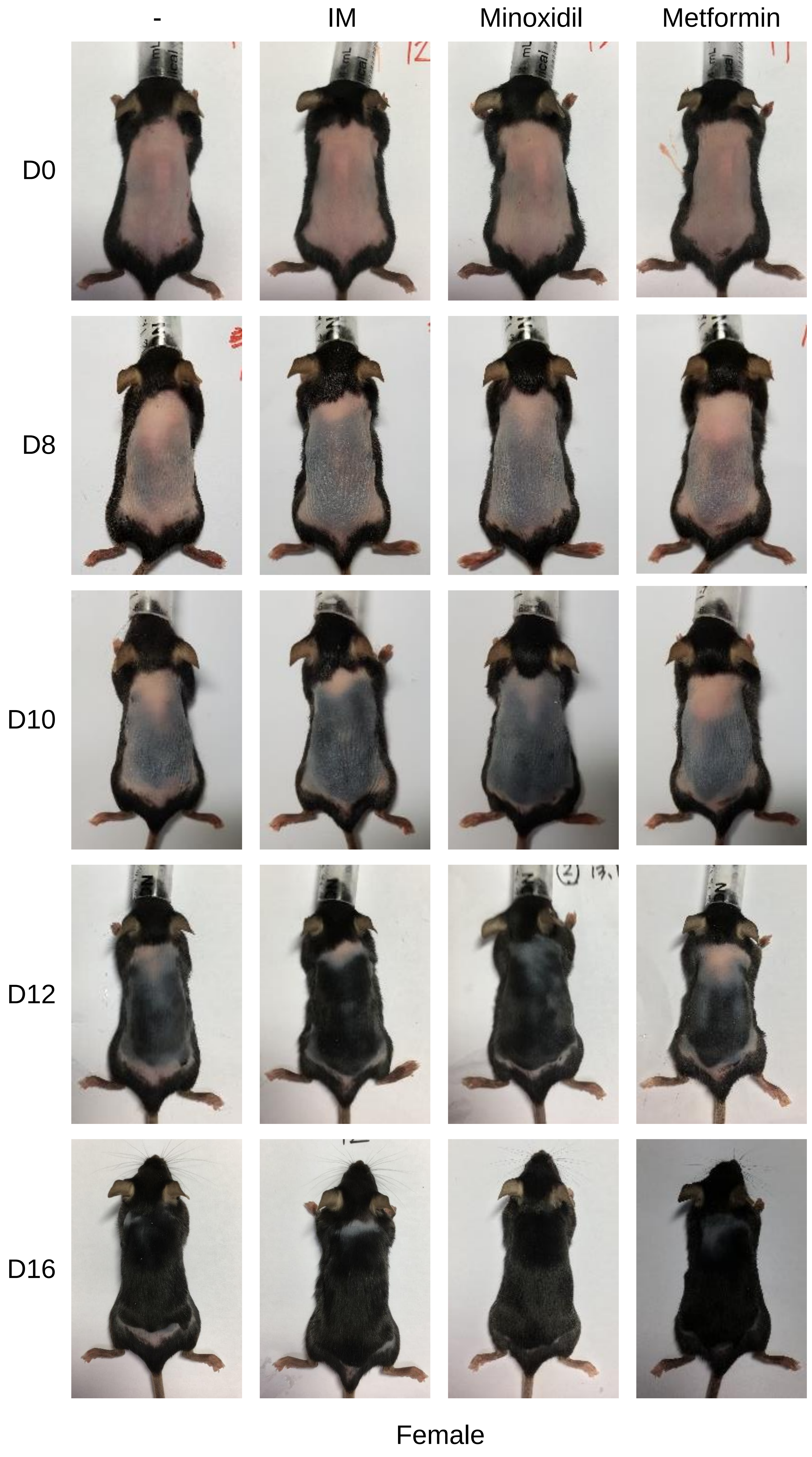

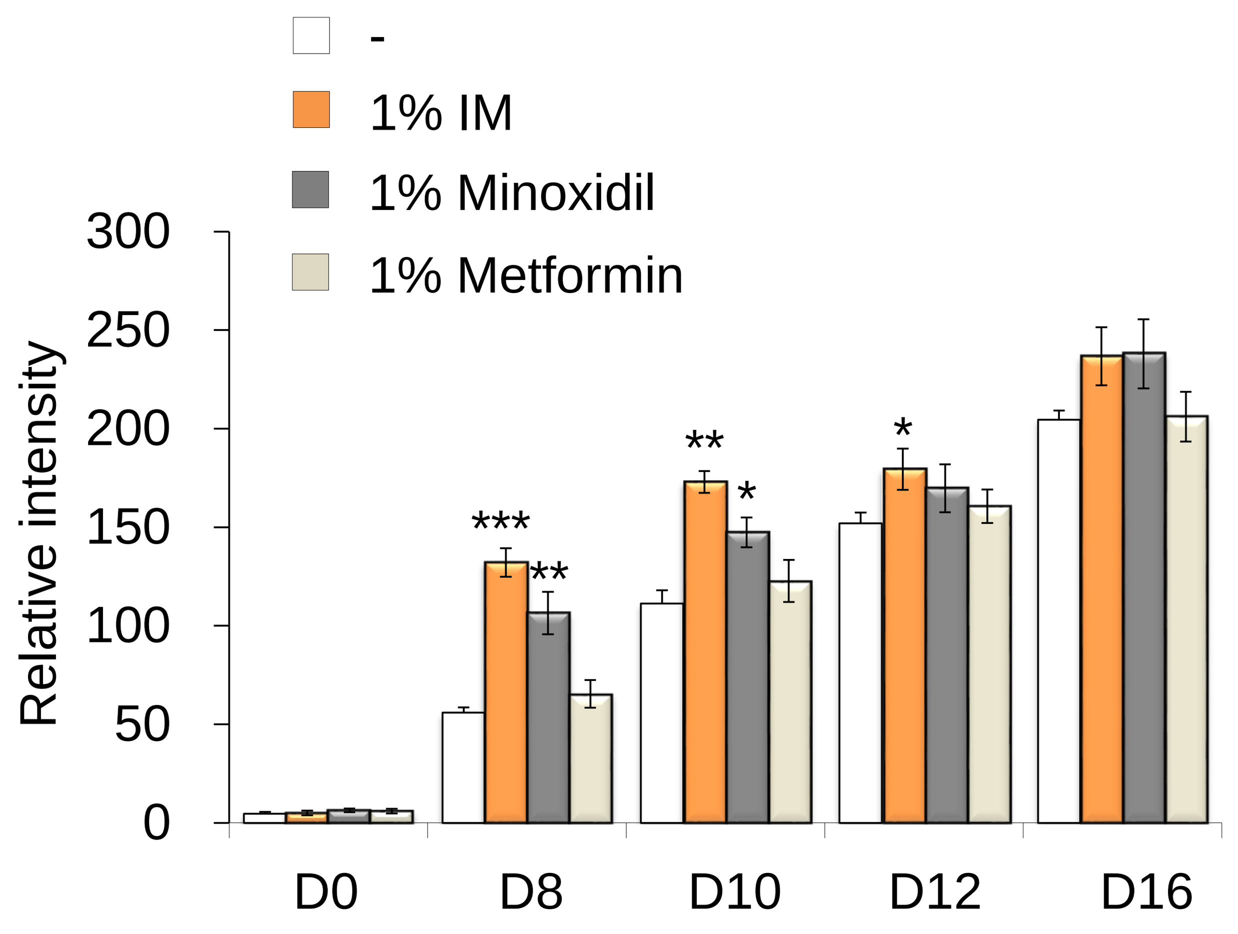

Supplementary Figure 11

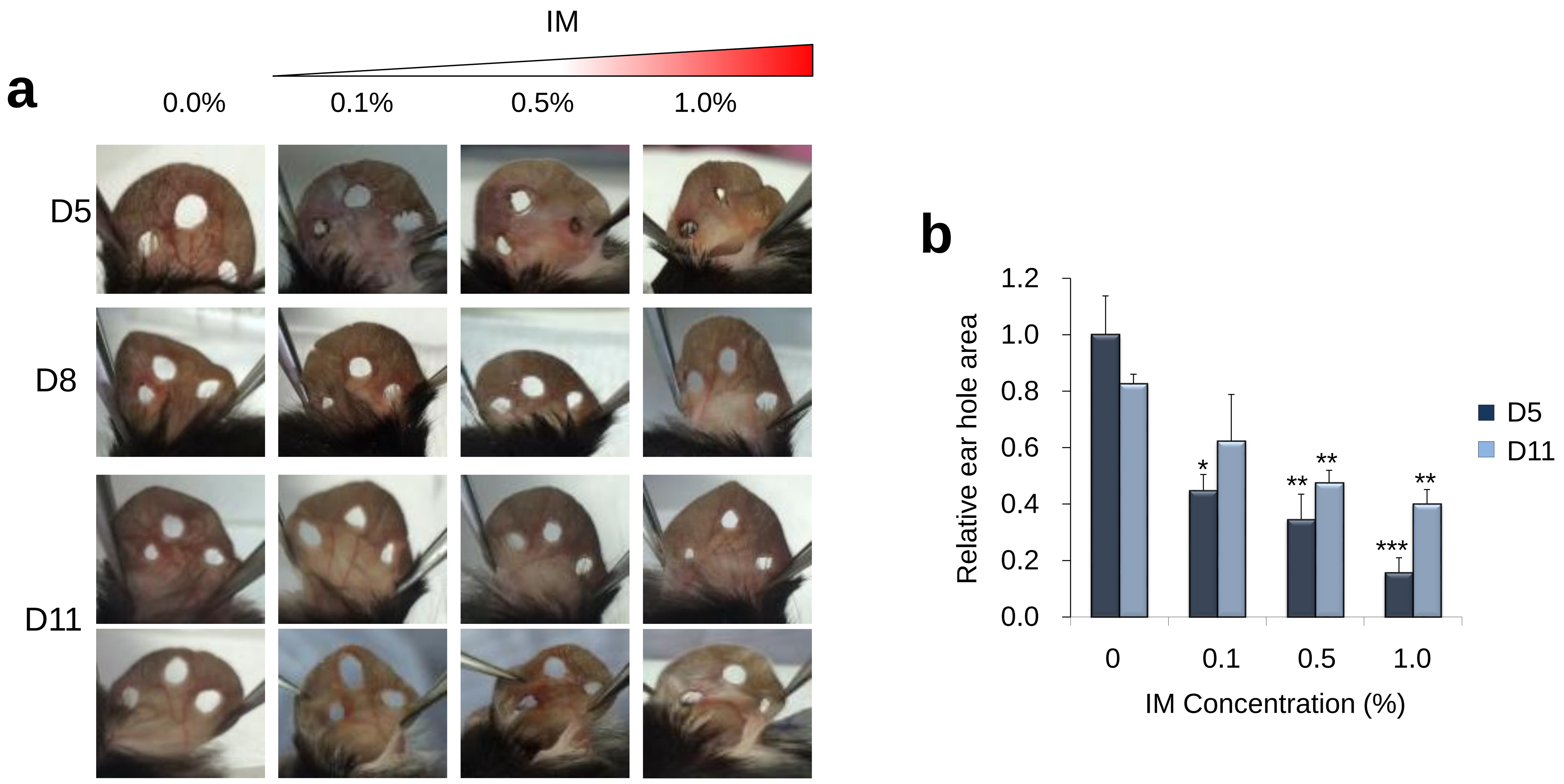

Supplementary Figure 12

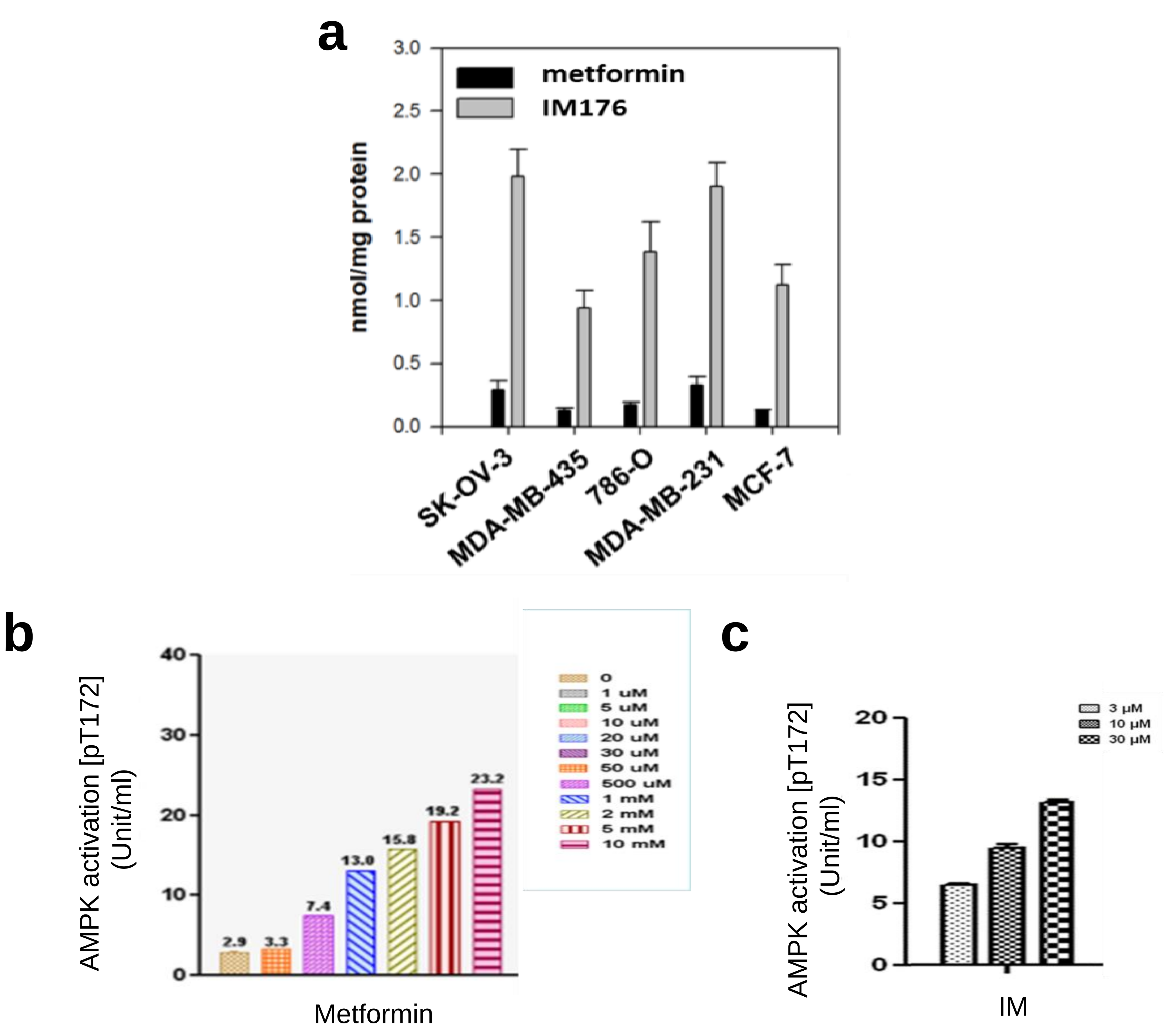

Supplement: Supplementary file 1 — Supplementary figures [file 12276_2018_185_MOESM1_ESM.pdf]
