# Supplementary material for: A novel and safe small molecule enhances hair follicle regeneration by facilitating metabolic reprogramming
Source: Exp Mol Med. 2018 Dec 6;50(12):1–15. doi: 10.1038/s12276-018-0185-z (PMC6283868; doi:10.1038/s12276-018-0185-z)
Supplement: Supplementary file 2 — Supplementary Figure Legends & Tables [file 12276_2018_185_MOESM2_ESM.docx]

**Supplementary Figure S1** Dose-response curves of cell viability in the presence of IM. Cell viability was compared in cell culture media with (a) 11.1 mM glucose or (b) 0.75 mM glucose using human SK-MEL-28 melanoma cells. Cells were treated with each concentration of IM for 72 hours. (c) MEFs and (d) OSKM-transduced MEFs cultured in conventional medium (25 mM glucose) were treated with each concentration of IM for 48 hours. Cell viability was determined as the cell number.

**Supplementary Figure S2** Optimal concentrations of IM in iPSC generation. (a) MEFs and (b) HFFs were reprogrammed with OSKM in the presence of the indicated concentrations of IM. Representative images of AP^+^ colonies (*each* *left*) and the total number of AP^+^ colonies (*each right*) are shown. (c) MEFs were transduced with each indicated reprogramming factor in the presence of 100 nM or 500 nM IM. Representative images of AP^+^ colonies and the total number of AP^+^ colonies on day 14 are shown. *p < 0.05; **p < 0.01; ***p < 0.001 (Student’s *t*-test).

**Supplementary Figure S3** Optimal concentrations of IM in ESC culture. (a) mESCs with or without LIF were cultured in the presence of the indicated concentrations of IM. Representative images of AP^+^ colonies (*left*) and the total number of AP^+^ colonies (*right*) on day 14 are shown. (b) hESCs were routinely maintained in the self-renewing condition (CM). hESCs cultured in the non-self-renewing condition (UM) were incubated with the indicated concentrations of IM for 6 days. Representative images of AP^+^ colonies (*left*) and relative AP expression (*right*) are shown. *p < 0.05; **p < 0.01; ***p < 0.001 (Student’s *t*-test).

**Supplementary Figure S4** Effects of biguanides on acquisition and maintenance of stemness. (a) MEFs and OG2-MEFs were reprogrammed with OSKM in the presence of the indicated concentrations of metformin. Representative images of AP^+^ colonies (*upper* *left*), Oct4-GFP^+^ colonies (*lower* *left*), and the total number of AP^+^ colonies (*right*) on day 14 are shown. The effect was compared with that of 10 nM IM. (b) MEFs were reprogrammed in the presence of the indicated concentrations of phenformin. Representative images of AP^+^ colonies (*left*) and the total number of AP^+^ colonies (*right*) on day 14 are shown. The effect was compared with that of 10 nM rotenone. (c) mESCs with or without LIF were cultured in the presence of the indicated concentrations of metformin for 4 days. Representative images of AP^+^ colonies (*left*) and the total number of AP^+^ colonies (*right*) are shown. The effect was compared with that of 10 nM IM. (d) hESCs were routinely maintained in the self-renewing condition (CM). hESCs cultured in the non-self-renewing condition (UM) were incubated with the indicated concentrations of metformin for 6 days. Representative images of AP^+^ colonies (*upper*) and relative AP expression (*lower*) are shown. The effect was compared with that of 10 nM IM. *p < 0.05; **p < 0.01; ***p < 0.001 (Student’s *t*-test).

**Supplementary Figure S5** IM promotes reprogramming kinetics. (a) OG2-MEFs were reprogrammed with OSKM in the absence and presence of IM. Representative morphologies on day 9 (*top*) and day 11 (*middle*) and Oct4-GFP^+^ colonies on day 11 of reprogramming (*bottom*) were shown. (b) The reprogramming kinetics were quantified by the number of days required for colony selection. (c) Representative immunofluorescence images of Nanog (green) and SSEA1 (red) staining (*left*) during reprogramming. DAPI staining was used to identify nuclei (blue). The numbers of fluorescent Nanog^+^ or SSEA1^+^ cell clusters were quantified (*right*). (d) ECAR was simultaneously measured in same sample of OCR (Figure 2b) by XFe96 Flux analyzer. (e) ChIP assays were performed on day 7 of reprogramming with or without IM treatment. Histone H3 lysine 9 dimethylation (H3K9me2) was precipitated and the *Nanog* and *Oct4* promoter loci was analyzed by real-time PCR. Input samples were used as a relative control. *p < 0.05; **p < 0.01; ***p < 0.001 (Student’s *t*-test). Scale bar = 100 μm.

**Supplementary Figure S6** Optimal concentrations of IM promote hair regrowth in mice. Dorsal skin hairs of 7-week-old C57BL/6 mice were depilated. Placebo control was applied on the left half of the dorsal skin, and various concentrations of IM were separately applied on the right half of the dorsal skin every day. Representative photos of mice in triplicate are shown on days 9, 11, 13, 15, and 17.

**Supplementary Figure S7** IM promotes hair regrowth in female mice. C57BL/6 female mice in telogen phase were depilated. Placebo control (-), 1% IM, or 1% minoxidil was topically applied every day. Representative photos of mice show that IM promoted skin color darkness and hair regrowth.

**Supplementary Figure S8** IM promotes hair regrowth in male mice. C57BL/6 male mice in telogen phase were depilated. Placebo control (-), 1% IM, or 1% minoxidil was topically applied every day. (a) and (b) show representative photos of repeated experiments with independent groups of male mice.

**Supplementary Figure S9** K15/PDH and Ki67/PDK expression in IM treated mice on day 20. (a) Immunohistochemistry of K15/PDH and Ki67/PDK in IM treated mouse skin on day 20. DAPI staining was used to identify nuclei (blue). Scale bar = 50 μm. (b) FACS analysis of K15/PDH and Ki67/PDK in single cells of each treated mouse skin on day 20.

**Supplementary Figure S10** Effect of metformin on hair regrowth. Placebo control (-), 1% IM, 1% minoxidil, or 1% metformin was topically applied every day to the dorsal skin of depilated C57BL/6 mice in telogen phase. Representative photos of mice show skin color darkness and hair regrowth on days 8, 10, 12, and 16 (*left*). The level of pigmentation was quantified by intensity of darkness in back skin of the same area (*right*). *p < 0.05; **p < 0.01; ***p < 0.001 (Student’s *t*-test).

**Supplementary Figure S11** IM facilitates pinnal tissue repair. Various concentrations of IM were topically applied to a punched wound area of the ear. (a) Representative ear images of each treated group on days 5, 8, and 11. (b) The hole area was measured, and the relative repair capacity was quantified based on wound closure. *p < 0.05; **p < 0.01; ***p < 0.001 (Student’s *t*-test).

**Supplementary Figure S12** IM exhibited improved cell penetration and then effectively activated AMPK compared with metformin. (a) Metformin or IM (10 μM) was treated for 30 minutes in each cell line having different level of OCT1 expression (SK-OV-3, MDA-MB-231>MDA-MB-435>786-O, MCF-7). Penetrated concentration of chemicals inside of the each cell line were quantified using LC-MS/MS. AMPK activation was quantified by phosphorylation of AMPKα (pT172) in the presence of various concentrations of (b) metformin and of (c) IM.

**Supporting Information Table 1**. List of primers used in this study.

| Gene | Primer (Forward) | Primer (Reverse) |
| --- | --- | --- |
| *mNDUS3* | CAAGCAGCTCTCAGCATTTG | CGCAGAGACAGCAGGTTGTA |
| *mATP5B* | GAGGGATTACCACCCATCCT | CATGATTCTGCCCAAGGTCT |
| *mHK2* | GGGACGACGGTACACTCAAT | GCCAGTGGTAAGGAGCTCTG |
| *mLDHA* | TGGCAGCCTCTTCCTTAAAA | CAGCTTGCAGTGTGGACTGT |
| *mNanog* | GGTCTTCCTGGTCCCCACAGTTTG | TGGGACTGGTAGAAGAATCAGGGC |
| *mRex1* | ACGAGTGGCAGTTTCTTCTTGGGA | TATGACTCACTTCCAGGGGGCACT |
| *mWnt1a* | GGTTTCTACTACGTTGCTACTGG | GGAATCCGTCAACAGGTTCGT |
| *mLef-1* | GCCACCGATGAGATGATCCC | TTGATGTCGGCTAAGTCGCC |
| *mGli-1* | CCAAGCCAACTTTATGTCAGGG | AGCCCGCTTCTTTGTTAATTTGA |
| *Versican* | TTTTACCCGAGTTACCAGACTCA | GGAGTAGTTGTTACATCCGTTGC |
| *mβ-actin* | AGCCATGTACGTAGCCATCC | CTCTCAGCTGTGGTGGTGAA |
| *Oct4 (ChIP)* | ATCCGAGCAACTGGTTTGTG | CAATCCCACCCTCTAGCCTT |
| *Nanog (ChIP* | TCTTTAGATCAGAGGATGCCCCCTAAGC | AAGCCTCCTACCCTACCCACCCCCTAT |

**Supporting Information Table 2**. . List of antibodies used in this study.

| Antibodies | Catalog No. | Company | Dilution |
| --- | --- | --- | --- |
| anti-Cytokeratin 15 | ab52816 | abcam | 1:100 |
| anti-β-catenine c | ab32572 | abcam | 1:200 |
| anti-β-catenine (FACS) | ab22656 | abcam | 1:500 |
| anti-Ki67 | ab15580 | abcam | 1:200 |
| anti-shh (IHC) | sc-9024 | Santacruz | 1:80 |
| anti-shh (FACS) | ab135240 | abcam | 1:300 |
| anti-PDK | sc-7140 | Santacruz | 1:50 |
| anti-PDH (rabbit IgG) | sc-292543 | Santacruz | 1:50 |
| anti-PDH (mouse IgG) | sc-377092 | Santacruz | 1:50 |
| Normal Rabbit IgG | #2729 | Cell signaling technology | 1:500 |
| anti-Histon H3 | #4620 | Cell signaling technology | 1:50 |
| anti-trimethyl-Histone H3 (Lys4) | 04-745 | Millipore | 1:200 |
| anti- trimethyl-Histone H3 (Lys27) | 07-449 | Millipore | 1:200 |
| anti-dimethyl-Histone H3 (Lys9) | 07-441 | Millipore | 1:200 |
| anti-NANOG | A300-397A | Bethyl Lab | 1:250 |
| anti-SSEA1 | MAB2155 | R&D Systems | 1:200 |
